# Structural Determination of Ruthenium Complexes Containing Bi-Dentate Pyrrole-Ketone Ligands

**DOI:** 10.3390/molecules23010159

**Published:** 2018-01-13

**Authors:** Ya-Wen Tsai, Yun-Fan Chen, Yong-Jie Li, Kuan-Hung Chen, Chia-Her Lin, Jui-Hsien Huang

**Affiliations:** 1 Department of Chemistry, National Changhua University of Education, Changhua 50058, Taiwan; s0225019@gmail.com (Y.-W.T.); evons44@gmail.com (Y.-F.C.); Jack100960503@gmail.com (Y.-J.L.); k19920922@yahoo.com.tw (K.-H.C.); 2 Department of Chemistry, Chung-Yuan Christian University, Chun-Li 320, Taiwan; chiaher@cycu.edu.tw

**Keywords:** ruthenium, pyrrole-ketone, cymene, ruthenium-hydride

## Abstract

A series of ruthenium compounds containing a pyrrole-ketone bidentate ligand, 2-(2′-methoxybenzoyl)pyrrole (**1**), have been synthesized and characterized. Reacting **1** with [(η^6^-cymene)RuCl_2_]_2_ and RuHCl(CO)(PPh_3_)_3_ generated Ru(η^6^-cymene)[C_4_H_3_N-2-(CO-C_6_H_4_-2-OMe)]Cl (**2**) and {RuCl(CO)(PPh_3_)_2_[C_4_H_3_N-2-(COC_6_H_4_-2-OMe)]} (**3**), respectively, in moderate yields. Successively reacting **2** with sodium cyanate and sodium azide gave {Ru(η^6^-cymene)[C_4_H_3_N-2-(CO-C_6_H_4_-2-OMe)]X} (**4**, X=OCN; **5**, X=N_3_) with the elimination of sodium chloride. Compounds **2**–**5** were all characterized by ^1^H and ^13^C-NMR spectra and their structures were also determined by X-ray single crystallography.

## 1. Introduction

Ruthenium compounds [1,2,3] containing varieties of ligands, such as cymene [4,5], phosphine [6] NHC [7], keto-amine [8,9], and Schiff-base [10,11], represent an important chemical series on the field of medicinal chemistry [12,13,14,15] and catalytic transfer hydrogenation [16,17,18] etc. Finely tuned ruthenium complexes can be achieved by changing the coordinating ligands. Therefore, by combining suitable ruthenium complexes with organic ligands and studying their structural geometries may shed some light on the understanding of the functions of these ruthenium complexes.

Among of these ruthenium compounds, [(η^6^-cymene)RuCl_2_]_2_ [19] and RuHCl(CO)(PPh_3_)_3_ [20] are two important starting materials for synthesizing corresponding ruthenium compounds due to their versatile applications [21,22,23,24]. Pyrrole represents an important heterocyclic compound for forming materials such as hemes and porphyrinc [25,26], ion-receptors [27,28], bio-active products [29,30,31], and light-harvesting pigments [32], etc. Its related compound, 2-(2′-methoxybenzoyl)pyrrole (**1**) [33,34,35], a multi-dentate ligand, was isolated from the mycelia extract [36] and exhibits bio-activity [37,38].

Here we report the synthesis and structural characterization of a series of ruthenium compounds containing the multidentate pyrrole-ketone ligand. The successive reacting of these ruthenium complexes with sodium salts of NaOCN and NaN_3_ is also reported. These compounds were structurally determined in order to understand their steric geometries for further applications.

## 2. Results and Discussion

### 2.1. Synthesis and Characterization

**Scheme 1:**
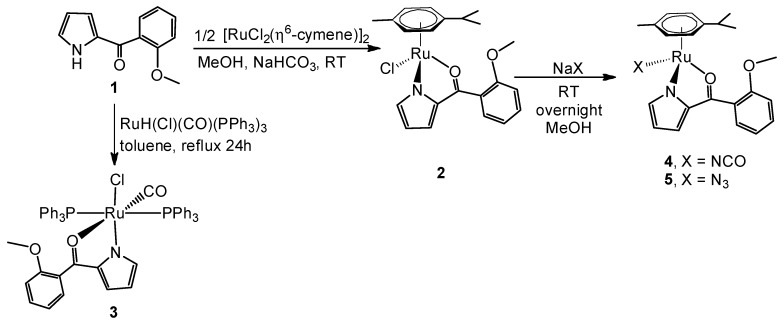
Synthesis of Compounds **2**–**5**.

A series of ruthenium compounds containing 2-(2′-methoxybenzoyl)pyrrole (**1**) was synthesized and characterized. A reaction scheme of **1** with [(η^6^-cymene)RuCl_2_]_2_ and RuHCl(CO)(PPh_3_)_3_ is shown in Scheme 1. Reacting [(η^6^-cymene)RuCl_2_]_2_ with two equivalents of **1** in methanol solution at room temperature in the presence of sodium bicarbonate gave a 32.0% yield of Ru(η^6^-cymene)[C_4_H_3_N-2-(CO-C_6_H_4_-2-OMe)]Cl (**2**). The ^1^H-NMR spectrum of **2** showed four doublets in the range of δ 5.62~5.35 representing the arene C*H* protons of the cymene fragment. Similarly, while reacting **1** with RuHCl(CO)(PPh_3_)_3_ in toluene under refluxing and work-up, the resulting product {RuCl(CO)(PPh_3_)_2_[C_4_H_3_N-2-(COC_6_H_4_-2-OMe)]} (**3**) was prepared in a 66.9% yield along with the elimination of one equivalent of hydrogen molecules. The ^1^H-NMR spectrum of **3** showed the pyrrole CH chemical shifts at δ 5.50, 5.59, and 6.65. Due to the weak hydrogen bonding, the isopropyl fragment showed two doublets in the ^1^H-NMR spectrum representing the slow C-C bond rotation of the isopropyl and the cymene phenyl ring. The ^13^C-NMR spectra of **3** showed two chemical shifts at δ 205.6 and 183.4, assigned as the carbon of C≡O and C=O, respectively [39,40]. The IR spectrum of **3** also showed a strong absorption band at 1942 cm^−1^ representing the stretching frequency of C≡O [41,42].

Successively replacing the chloride anion of **2** with sodium salts of NaX in methanol resulted in new ruthenium compounds {Ru(η^6^-cymene)[C_4_H_3_N-2-(CO-C_6_H_4_-2-OMe)]X} (**4**, X=OCN; **5**, X=N_3_). Compounds **4** and **5** were both characterized by ^1^H and ^13^C-NMR spectra and showed patterns similar to those of Compound **2**, indicating that the electronic properties around the ruthenium atom was not affected by the coordinated mono-anionic ligands.

### 2.2. Molecular Geometries of Compounds ***2***–***5***

**Figure 1:**
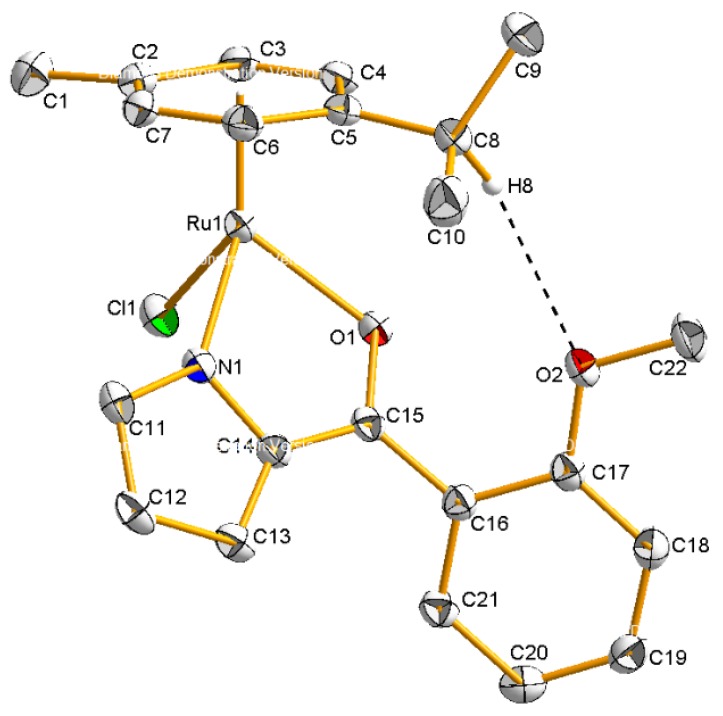
The molecular geometry of Compound **2**. The thermal ellipsoids were drawn at 50% probability and all the hydrogen atoms except the methine proton on the cymene ring were omitted for clarity. The H8-O2 hydrogen bonding is shown.

**Figure 2:**
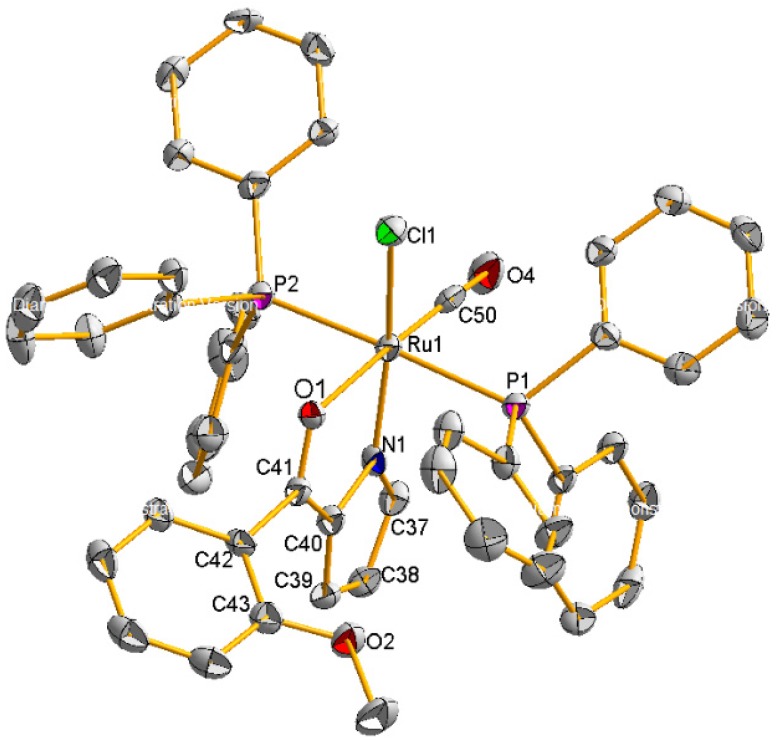
The molecular geometry of Compound **3**. The thermal ellipsoids were drawn at 50% probability and all the hydrogen atoms were omitted for clarity.

**Figure 3:**
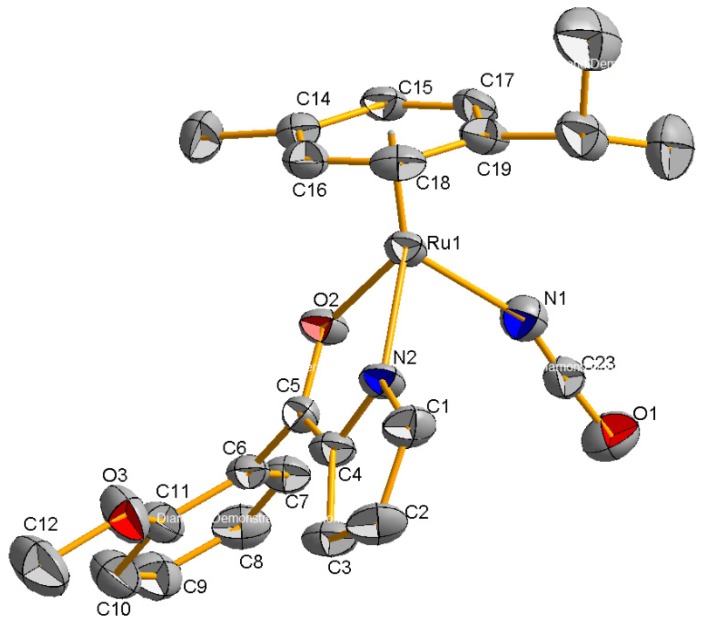
The molecular geometry of Compound **4**. The thermal ellipsoids were drawn at 50% probability and all the hydrogen atoms were omitted for clarity.

**Figure 4:**
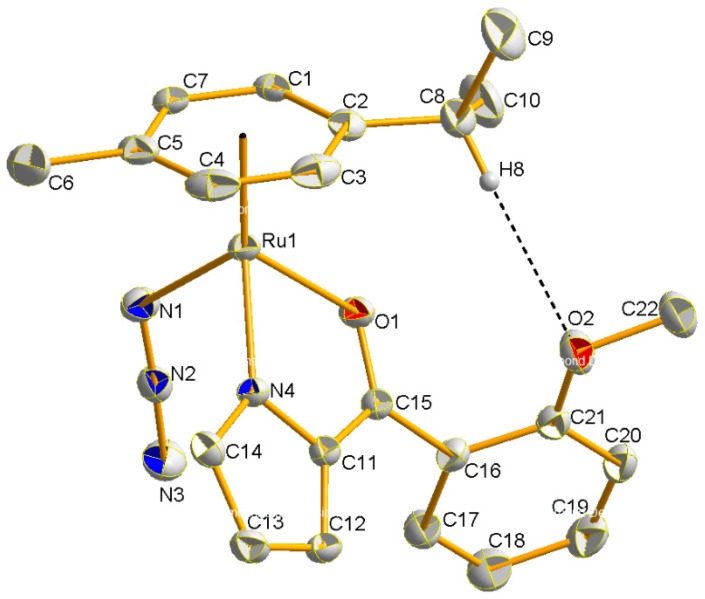
The molecular geometry of Compound **5**. The thermal ellipsoids were drawn at 50% probability and all the hydrogen atoms except the methine proton on the cymene ring were omitted for clarity. The H8-O2 hydrogen bonding is shown.

Single-crystals of Compounds **2**–**5** for X-ray diffraction analysis were obtained from either methanol or THF saturated solution at −20 °C. The summary of the X-ray crystal data and selected bond lengths and angles are shown in Table 1 and Table 2, respectively. The molecular geometries of **2**–**5** are depicted in Figure 1, Figure 2, Figure 3, Figure 4. The molecular geometry of **2** could be described as a three-legged piano stool with the nitrogen atom of the pyrrole, the oxygen atom of the carbonyl atoms, and the chloride atom forming the three legs and the cymene ring acting as the stool plane. The bond length of the ruthenium atom to the center of the cymene ring is 1.6598(1) Å, relatively close to the bond lengths of published ruthenium-cymene compounds [41,42]. It is interesting to note that the methine proton (H8) of the cymene ring was close to the oxygen atom (O2) of the methoxy group of ligand **1**, with a bond length of 2.511 Å, which is assumed to be a weak hydrogen bonding between the H8 and O2 atoms [43].

The molecular geometry of **3** is shown in Figure 2 and the THF molecule was omitted for clarity. It can be described as a distorted octahedral with three axes of Cl(1)-Ru(1)-N(1), O(1)-Ru(1)-C(50), and P(1)-Ru(1)-P(2) with angles of 166.70(14)°, 172.9(2)°, and 177.38 (5)°, respectively. The ligand **1** binds to the ruthenium atom in an acute chelating angle of N(1)-Ru(1)-O(1) at 78.32(16)°. The bond lengths of Ru(1)-C(50) and C(50)-O(4) are 1.845(7) Å and 1.128(8) Å, respectively, indicating a slight back bonding of the ruthenium atom to the carbonyl and the longer C≡O bond. The results are in agreement with reported trans-RuCl(CO)(PPh_3_)_2_(bidentate) structures [44,45,46,47,48]. 

The molecular geometry of **4** can also be described as a three-legged piano stool geometry, as shown in Figure 3. The bond length from the ruthenium atom to the center of the cymene ring is at ca. 1.667 Å. The ambi-dentate NCO ligand was bound to the ruthenium atom at the N-end. Similar ruthenium cymene-NCO structures have been reported in the literature [49,50]. The bond lengths of ruthenium to the cyanate-N atom as well as N=C and C=O are all consistent with the results in the literature.

The pale orange crystals of **5** were obtained from a saturated methanol solution at −20 °C. The molecular structure of **5** is shown in Figure 4 and its geometry is similar to that of **2**. It can also be described as a three-legged piano stool geometry with the azide and pyrrolyl nitrogen atoms and carboxyl oxygen atom taking the three leg positions and the cymene acting as the planar surface. The bond length of O(2)-H(8) was ca. 2.868 Å, indicating a very weak or no hydrogen bonding effect. Ru(cymene)azido compounds have been reported in the literature [51,52,53,54]. For **5**, the bond lengths of Ru(1)-N(1), N(1)-N(2), and N(2)-N(3) were 2.109(2) Å, 1.204(3) Å, and 1.157(3) Å, respectively and these data are consistent that reported in the literature.

## 3. Experimental Section

### 3.1. General Consideration

All reactions were performed under a nitrogen atmosphere using standard Schlenk techniques or in a glove box. Toluene was dried by refluxing over sodium benzophenone ketyl. CH_2_Cl_2_ was dried over P_2_O_5_. All solvents were distilled and stored in solvent reservoirs that contained 4-Å molecular sieves and were purged with nitrogen. The ^1^H and ^13^C-NMR spectra were recorded using a Bruker Avance 300 spectrometer and the chemical shifts were recorded in ppm relative to the residual protons of CDCl_3_ (δ = 7.24, 77.0 ppm). Elemental analyses were performed using a Heraeus CHN-OS Rapid Elemental Analyzer at the Instrument Center of the NCHU. Ligands [33,34,35] **1** and [Ru(η^6^-p-cymene)Cl_2_]_2_ were prepared using a modified procedure [19].

#### 3.1.1. Synthesis of the Ligand [C_4_H_3_NH-2-(CO-C_6_H_4_-2-OMe)] (**1**)

A Schlenk flask charged with pyrrole (0.38 g, 5.7 mmol) and 15 mL of toluene was added to equimolar MeMgBr (3.0 M, 1.9 mL) at 0 °C. The resulting solution was then refluxing for 30 min under nitrogen. The color changed from bright yellow back to orange. Methoxybenzoyl chloride (0.97 g, 5.7 mmol) was then added to the resulting solution at room temperature and the solution color changed back to yellow again. Ammonium chloride water solution was added to the solution after 30 min and the combing solution was extracted with diethyl ether (10 mL × 3). The extraction was dried over MgSO_4_ and volatiles were removed to give ligand 1 in a 63.0% yield (0.727 g). ^1^H-NMR (CDCl_3_): 3.80 (s, 3H, O*Me*), 6.24 (m, 1H, pyr C*H*), 6.62 (m, 1H, pyr C*H*), 6.99 (m, 2H, phenyl C*H*), 7.10 (m, 1H, pyr C*H*), 7.42 (m, 2H, phenyl C*H*), 10.12 (s, 1H, pyr N*H*).

#### 3.1.2. Synthesis of Compound {Ru(η^6^-cymene)[C_4_H_3_N-2-(CO-C_6_H_4_-2-OMe)]Cl} (**2**)

A methanol solution (10 mL) of **1** (0.20 g, 0.99 mmol) and [Ru(η^6^-*p*-cymene)Cl_2_]_2_ (0.304 g, 0.50 mmol) in a 50 mL of flask was stirred for 1 h at room temperature. Sodium bicarbonate then was added into the solution and stirred for 3 h. The volatiles were removed under vacuum and the solid was extracted with methylene chloride and then solvent was removed again to give crude product of **2**. The product was recrystallized from a methanol solution to generate 0.149 g of red brown crystals in a 32.0% yield. ^1^H-NMR (CDCl_3_): 1.21 (d, 3H, CH*Me*_2_), 1.23 (d, 3H, CH*Me*_2_), 2.30 (s, 3H, *Me*), 2.81 (sept, *J*_HH_ = 7.2 Hz, 1H, C*H*Me_2_), 3.77 (s, 3H, O*Me*), 5.35 (d, *J*_HH_ = 6.0 Hz, 1H, cymene C*H*), 5.40 (d, *J*_HH_ = 6.0 Hz, 1H, cymene C*H*), 5.55 (d, *J*_HH_ = 5.7 Hz, 1H, cymene C*H*), 5.62 (d, *J*_HH_ = 5.7 Hz, 1H, cymene C*H*), 6.37 (m, 1H, pyr C*H*), 6.79 (m, 1H, pyr C*H*), 7.36 (m, 5H, phenyl C_6_H_4_ + pyr C*H*). ^13^C-NMR (CDCl_3_): 18.9 (cymene *Me*), 22.1 (cymene CH*Me*_2_), 22.8 (cymene CH*Me*_2_), 31.9 (cymene *C*HMe_2_), 55.9 (O*Me*), 79.3 (cymene *C*H), 81.8 (cymene *C*H), 82.0 (cymene *C*H), 83.5 (cymene *C*H), 98.4 (cymene *C_ipso_*), 100.5 (cymene *C_ipso_*), 111.8 (pyr *C*H), 116.1 (pyr *C*H), 120.4 (phenyl *C*H), 123.4 (phenyl *C*H), 125.5 (pyr *C_ipso_*), 131.4 (phenyl *C*H), 132.1 (phenyl *C*H), 141.8 (phenyl *C_ipso_*), 143.1 (pyr *C*H), 157.7 (phenyl *C_ipso_*), 186.7 (*C=*O). Anal. Calcd for C_22_H_24_ClNO_2_Ru: C, 56.11; H, 5.14; N, 2.97. Found: C, 56.08; H, 5.15; N, 3.36.

#### 3.1.3. Synthesis of Compound {RuCl(CO)(PPh_3_)_2_[C_4_H_3_N-2-(COC_6_H_4_-2-OMe)]} (**3**)

A toluene solution (10 mL) of **1** (0.08 g, 0.40 mmol) and RuHCl(CO)(PPh_3_)_3_ (0.378 g, 0.40 mmol) in a 50 mL of flask was refluxed for 24 h under nitrogen. The volatiles were removed under vacuum and residue was washed with heptane to remove excess of PPh_3_. The resulting solid was recrystallized from a saturated THF solution at −20 °C to give 0.24 g of yellow crystals in a 66.9% yield. ^1^H-NMR (CDCl_3_): 3.31 (s, 3H, O*Me*), 5.50 (t, 1H, pyr C*H*), 5.59 (m, 1H, pyr C*H*), 6.20 (m, 1H, phenyl C*H*), 6.40 (m, 1H, phenyl C*H*), 6.65 (m, 2H, pyr + phenyl C*H*), 7.30 (m, 31H, PPh_3_ C*H*+ phenyl C*H*). ^13^C-NMR (CDCl_3_): 55.0 (O*Me*), 110.3, 117.3, 119.7, 123.0, 125.4, 126.6, 127.8, 127.9, 128.0, 128.3, 128.6, 128.7, 129.2, 129.5, 130.5, 130.9, 131.0, 131.2, 131.5, 132.2, 134.5, 134.6, 134.7,141.3, 142.6, 156.3, 183.4 (*C*=O), 205.6 (*C*≡O). Anal. Calcd for C_49_H_40_NO_3_P_2_ClRu: C, 66.13; H, 4.53; N, 1.57. Found: C, 66.01; H, 4.86; N, 1.53.

#### 3.1.4. Synthesis of Compound {Ru(η^6^-cymene)[C_4_H_3_N-2-(CO-C_6_H_4_-2-OMe)](OCN)} (**4**)

A 25 mL Schlenk flask charged with **2** (0.117 g, 0.25 mmol), excess sodium cyanate and methanol (10 mL) was stirred at room temperature for overnight. Methanol was removed under vacuum and residue was extracted with methylene chloride (10 mL × 3). The methylene chloride was removed and residue was recrystallized from a methanol solution at −20 °C to yield 0.045 g of orange crystals of **4** (38.1% yield). ^1^H-NMR (CDCl_3_): 1.20 (d, 3H, CH*Me*_2_), 1.22 (d, 3H, CH*Me*_2_), 2.25 (s, 3H, *Me*), 2.74 (sept, *J*_HH_ = 6.9 Hz,1H, C*H*Me_2_), 3.77 (s, 3H, O*Me*), 5.32 (d, 2H, *J*_HH_ = 6.3 Hz, cymene C*H*), 5.57 (d, *J*_HH_ = 5.4 Hz, 1H, cymene C*H*), 5.62 (d, *J*_HH_ = 5.4 Hz, 1H, cymene C*H*), 6.37 (m, 1H, pyr C*H*), 6.78 (m, 1H, pyr C*H*), 7.38 (m, 4H, phenyl C_6_H_4_ C*H*), 7.62 (t, 1H, pyr C*H*). Anal. Calcd for C_23_H_24_N_2_O_3_Ru: C, 57.70; H, 5.06; N, 5.86. Found: C, 57.70; H, 5.18; N, 6.04.

#### 3.1.5. Synthesis of Compound {Ru(η^6^-cymene)[C_4_H_3_N-2-(CO-C_6_H_4_-2-OMe)](N_3_)} (**5**)

A 25 mL Schlenk flask charged with **2** (0.047 g, 0.10 mmol), excess sodium azide, and methanol (10 mL) was stirred at room temperature for overnight. Methanol was removed under vacuum and residue was extracted with methylene chloride (10 mL × 3). The methylene chloride was removed and residue was recrystallized from a methanol solution at −20°C to yield 0.026 g of orange crystals of **5** (53.8% yield). ^1^H-NMR (CDCl_3_): 1.23 (d, 3H, CH*Me*_2_), 1.25 (d, 3H, CH*Me*_2_), 2.27 (s, 3H, *Me*), 2.78 (sept, *J*_HH_ = 6.9 Hz,1H, C*H*Me_2_), 3.80 (s, 3H, O*Me*), 5.32 (m, 2H, cymene C*H*), 5.53 (d, *J*_HH_ = 6.0 Hz, 1H, cymene CH), 5.62 (d, *J*_HH_ = 6.0 Hz, 1H, cymene C*H*), 6.43 (t, 1H, pyr C*H*), 6.82 (m, 1H, pyr C*H*), 7.38 (m, 5H, phenyl C_6_*H*_4_ + pyr C*H*). ^13^C-NMR (CDCl_3_): 18.4 (cymene *Me*), 22.5 (cymene CH*Me*_2_), 22.8 (cymene CH*Me*_2_), 31.2 (cymene *C*HMe_2_), 55.9 (O*Me*), 78.9 (cymene *C*H), 81.7 (cymene *C*H), 82.4 (cymene *C*H), 84.2 (cymene *C*H), 98.7 (arene C), 100.5 (*C_ipso_*),111.7 (pyr *C*H), 116.2 (pyr *C*H), 120.5 (phenyl *C*H), 123.7 (phenyl *C*H), 125.2 (*C_ipso_*), 131.6 (phenyl *C*H), 132.1 (phenyl *C*H), 142.1 (*C_ipso_*), 143.2 (pyr *C*H), 157.7 (*C_ipso_*), 187.2 (*C*=O). Anal. Calcd for C_22_H_24_N_4_O_2_Ru: C, 55.34; H, 5.07; N, 11.73. Found: C, 55.32; H, 5.09; N, 11.75.

### 3.2. X-ray Crystallography

Suitable crystals of Compounds **2**–**5** were attached to a fine glass fiber and mounted in goniostat for data collection. Data collections were performed at 150 K under liquid nitrogen vapor for all compounds. Data were collected on a Bruker SMART CCD diffractometer with graphite monochromated Mo-Kα radiation. No significant crystal decay was found. Data were corrected for absorption empirically by means of ψ scans. All non-hydrogen atoms were refined with anisotropic displacement parameters. For all the structures, the hydrogen atom positions were calculated and they were constrained to idealized geometries and treated as rigid where the H atom displacement parameter was calculated from the equivalent isotropic displacement parameter of the bound atom. An absorption correction was performed with the program SADABS [55] and the structures of both complexes were determined by direct methods procedures in SHELXS [56] and refined by full-matrix least-squares methods, on *F^2^*’s, in SHELXL [57]. All the relevant crystallographic data and structure refinement parameters are summarized in Table 1.

## 4. Conclusions

We have successfully employed a bidentate pyrrole-ketone ligand with ruthenium compounds to form a series of Compounds **2**–**5** and their structures were determined by X-ray single crystallography. A preliminary test of hydrogen transfer reactions of acetophenone and isopropyl alcohol using these ruthenium compounds showed low conversion. We are currently using Compounds **2**–**5** to investigate the catalytic activities of hydrogen transfer reactions toward varieties of ketones and alcohols and the hydroaminations of inter- and intra-molecular alkenes and alkynes.

## Figures and Tables

**Table 1:** The summary of X-ray crystal data for Compounds **2**–**5.**

	2	3 THF	4	5
formula	C_22_H_24_ClNO_2_Ru	C_53_H_48_ClNO_4_P_2_Ru	C_23_H_24_N_2_O_3_Ru	C_22_H_24_N_4_O_2_Ru
FW	470.94	961.38	477.51	477.52
*T* [K]	150(2)	150(2)	150(2)	150(2)
crystal system	Monoclinic	Monoclinic	Monoclinic	Monoclinic
space group	P2_1_/n	P2_1_/n	P2_1_/c	P2_1_/c
*a* [Å]	10.4657(4)	10.2279(5)	10.157(5)	17.3849(14)
*b* [Å]	9.1505(3)	17.5822(9)	15.836(7)	9.2423(7)
*c* [Å]	21.1478(8)	25.9234(13)	14.019(8)	13.8157(11)
α [°]	90	90	90	90
β [°]	99.097(2)	97.693(3)	105.44(3)	112.379(4)
γ [°]	90	90	90	90
*V* [Å^3^]	1999.78(13)	4619.8(4)	2173.5(19)	2052.7(3)
Z	4	4	4	4
*ρ*_c_ [Mg m^−3^]	1.564	1.382	1.465	1.545
μ [mm^−1^]	0.934	0.513	0.748	0.787
*F*(000)	960	1824	980	976.0
rflns collected	28,629	49,633	26,439	18,124
independent rflns	5176 [R_int_ = 0.0263]	8132 [R_int_ = 0.0979]	5380 [R_int_ = 0.0893]	5243 [R_int_ = 0.0298]
data/restraints/parameters	5176/0/248	8132/0/560	5380/0/266	5243/0/266
goodness-of-fit on *F*^2^	1.027	0.880	0.968	1.067
*R*_1_, *wR*_2_ (*I* > 2σ(*I*))	R_1_ = 0.0208	R_1_ = 0.0591	R_1_ = 0.0568	R_1_ = 0.0306
	wR_2_ = 0.0494	wR_2_ = 0.1682	wR_2_ = 0.1449	wR_2_ = 0.0783
*R*_1_, *wR*_2_ (all data)	R_1_ = 0.0260	R_1_ = 0.0879	R_1_ = 0.0890	R_1_ = 0.0356
	wR_2_ = 0.0520	wR_2_ = 0.1925	wR_2_ = 0.1724	wR_2_ = 0.0810
largest diff. peak, hole [*e*Å^−3^]	0.406 and −0.367	0.811 and −0.925	1.071 and −2.014	0.870 and −0.699

**Table 2:** Selected bond lengths (Å) and angles (°) for Compounds **2**–**5**.

**2**			
Ru(1)-Cl(1)	2.4092(5)	Ru(1)-N(1)	2.060(1)
Ru(1)-O(1)	2.103(1)	Ru(1)-Cymene	1.6598(1)
O(1)-C(15)	1.273(2)	N(1)-Ru(1)-O(1)	77.09(5)
Cl(1)-Ru(1)-N(1)	85.41(4)	Cl(1)-Ru(1)-O(1)	85.53(3)
**3**			
Ru(1)-C(50)	1.845(7)	Ru(1)-N(1)	2.038(5)
Ru(1)-O(1)	2.139(4)	Ru(1)-P(1)	2.4058(15)
O(1)-C(15)	1.273(2)	Ru(1)-P(2)	2.3908(15)
Ru(1)-Cymene	1.6598(1)	Ru(1)-Cl(1)	2.4185(15)
O(1)-C(41)	1.280(7)	C(50)-O(4)	1.128(8)
C(50)-Ru(1)-O(1)	172.9(2)	P(2)-Ru(1)-P(1)	177.38(5)
N(1)-Ru(1)-Cl(1)	166.70(14)	N(1)-Ru(1)-O(1)	78.32(16)
**4**			
Ru(1)-N(1)	2.070(5)	Ru(1)-N(2)	2.057(3)
Ru(1)-O(2)	2.124(3)	Ru(1)-Cymene	1.6674(7)
O(2)-C(5)	1.278(5)	N(1)-C(23)	1.155(6)
O(1)-C(23)	1.218(6)	Ru(1)-N(1)-C(23)	156.9(4)
N(1)-Ru(1)-N(2)	84.53(16)	N(2)-Ru(1)-O(2)	77.03(13)
N(1)-Ru(1)-O(2)	83.61(16)	N(1)-C(23)-O(1)	176.3(6)
**5**			
Ru(1)-N(1)	2.109(2)	Ru(1)-N(2)	2.0640(19)
Ru(1)-O(1)	2.1114(15)	Ru(1)-Cymene	1.6610(2)
O(1)-C(15)	1.276(3)	N(1)-N(2)	1.204(3)
N(2)-N(3)	1.157(3)	N(4)-Ru(1)-O(1)	77.08(7)
N(1)-Ru(1)-N(4)	86.02(8)	N(1)-Ru(1)-O(1)	86.62(7)
Ru(1)-N(1)-N(2)	120.43(17)	N(1)-N(2)-N(3)	176.7(3)

## Supplementary Materials

Tables for crystal data including H-bonding. Crystallographic data for the structures reported in this paper have been deposited with the Cambridge Crystallographic Data Centre as supplementary publications nos. CCDC-1567212-5 (Compounds **2**–**5**). Copies of the data can be obtained free of charge on application to CCDC, 12 Union Road, Cambridge CB2 1EZ, UK (fax: +44-1223-336-033; e-mail: deposit@ccdc.cam.ac.uk or www: http://www.ccdc.cam.ac.uk).
