# Supplementary material for: Structural Determination of Ruthenium Complexes Containing Bi-Dentate Pyrrole-Ketone Ligands
Source: Molecules. 2018 Jan 13;23(1):159. doi: 10.3390/molecules23010159 (PMC6016999; doi:10.3390/molecules23010159)
Supplement: Supplementary file 1 [file molecules-23-00159-s001.pdf]

Table 1. Crystal data and structure refinement for 2.

Identification code	2	
Empirical formula	C22 H24 Cl N O2 Ru	
Formula weight	470.94	
Temperature	150(2) K	
Wavelength	0.71073 Å	
Crystal system	Monoclinic	
Space group	P 21/n	
Unit cell dimensions	a = 10.4657(4) Å	α= 90°.
	b = 9.1505(3) Å	β= 99.097(2)°.
	c = 21.1478(8) Å	γ = 90°.
Volume	1999.78(13) Å^3	
Z	4	
Density (calculated)	1.564 Mg/m^3	
Absorption coefficient	0.934 mm^{-1}	
F(000)	960	
Crystal size	0.18 x 0.14 x 0.12 mm^3	
Theta range for data collection	1.95 to 28.75°.	
Index ranges	-14<=h<=14, -12<=k<=12, -28<=l<=25	
Reflections collected	28629	
Independent reflections	5176 [R(int) = 0.0263]	
Completeness to theta = 28.75°	99.8 %	
Absorption correction	Semi-empirical from equivalents	
Max. and min. transmission	0.8962 and 0.8499	
Refinement method	Full-matrix least-squares on F^2	
Data / restraints / parameters	5176 / 0 / 248	
Goodness-of-fit on F^2	1.027	
Final R indices [I>2sigma(I)]	R1 = 0.0208, wR2 = 0.0494	
R indices (all data)	R1 = 0.0260, wR2 = 0.0520	
Largest diff. peak and hole	0.406 and -0.367 e.Å^{-3}	

Table 2. Atomic coordinates (x 10^4) and equivalent isotropic displacement parameters (Å^{2}x 10^3) for 2. U(eq) is defined as one third of the trace of the orthogonalized U^{ij} tensor.

	x	y	z	U(eq)
Ru(1)	5663(1)	3196(1)	995(1)	20(1)
Cl(1)	7674(1)	4514(1)	1072(1)	29(1)
C(7)	5712(2)	842(2)	869(1)	27(1)
C(8)	2495(2)	3037(2)	649(1)	27(1)
C(5)	3781(2)	2389(2)	547(1)	24(1)
C(6)	4437(2)	1288(2)	942(1)	26(1)
C(4)	4423(2)	3004(2)	60(1)	26(1)
C(3)	5674(2)	2572(2)	-10(1)	27(1)
C(1)	7726(2)	1126(2)	349(1)	37(1)
C(9)	1416(2)	2649(2)	99(1)	37(1)
C(10)	2112(2)	2640(3)	1286(1)	43(1)
C(2)	6351(2)	1486(2)	402(1)	28(1)
N(1)	5962(1)	3290(1)	1981(1)	21(1)
O(1)	4848(1)	5222(1)	1180(1)	22(1)
C(16)	4405(2)	6982(2)	1947(1)	21(1)
C(15)	4955(1)	5577(2)	1768(1)	20(1)
C(14)	5566(1)	4590(2)	2228(1)	21(1)
C(12)	6457(2)	3218(2)	3057(1)	27(1)
C(13)	5865(2)	4552(2)	2902(1)	25(1)
C(11)	6488(1)	2472(2)	2478(1)	24(1)
C(17)	3115(2)	7365(2)	1706(1)	22(1)
C(21)	5139(2)	7919(2)	2379(1)	24(1)
C(18)	2596(2)	8650(2)	1907(1)	26(1)
C(20)	4631(2)	9220(2)	2567(1)	28(1)
C(19)	3360(2)	9570(2)	2332(1)	28(1)
O(2)	2434(1)	6391(1)	1297(1)	29(1)
C(22)	1089(2)	6673(2)	1098(1)	35(1)

Table 3. Bond lengths [Å] and angles [°] for 2.

Ru(1)-N(1)	2.0601(13)
Ru(1)-O(1)	2.1030(11)
Ru(1)-C(6)	2.1585(16)
Ru(1)-C(7)	2.1724(16)
Ru(1)-C(5)	2.1753(15)
Ru(1)-C(4)	2.1943(15)
Ru(1)-C(2)	2.1960(16)
Ru(1)-C(3)	2.2028(15)
Ru(1)-Cl(1)	2.4092(4)
C(7)-C(2)	1.406(2)
C(7)-C(6)	1.427(2)
C(8)-C(10)	1.510(3)
C(8)-C(5)	1.517(2)
C(8)-C(9)	1.529(2)
C(5)-C(6)	1.415(2)
C(5)-C(4)	1.432(2)
C(4)-C(3)	1.397(2)
C(3)-C(2)	1.434(2)
C(1)-C(2)	1.498(2)
N(1)-C(11)	1.3358(19)
N(1)-C(14)	1.3892(19)
O(1)-C(15)	1.2728(18)
C(16)-C(21)	1.392(2)
C(16)-C(17)	1.410(2)
C(16)-C(15)	1.482(2)
C(15)-C(14)	1.406(2)
C(14)-C(13)	1.411(2)
C(12)-C(13)	1.384(2)
C(12)-C(11)	1.408(2)
C(17)-O(2)	1.3621(19)
C(17)-C(18)	1.390(2)
C(21)-C(20)	1.387(2)
C(18)-C(19)	1.388(2)
C(20)-C(19)	1.382(2)

O(2)-C(22)	1.4277(19)
N(1)-Ru(1)-O(1)	77.09(4)
N(1)-Ru(1)-C(6)	94.59(6)
O(1)-Ru(1)-C(6)	117.86(5)
N(1)-Ru(1)-C(7)	99.37(6)
O(1)-Ru(1)-C(7)	156.24(5)
C(6)-Ru(1)-C(7)	38.48(6)
N(1)-Ru(1)-C(5)	115.91(6)
O(1)-Ru(1)-C(5)	90.81(5)
C(6)-Ru(1)-C(5)	38.11(6)
C(7)-Ru(1)-C(5)	69.35(6)
N(1)-Ru(1)-C(4)	152.74(6)
O(1)-Ru(1)-C(4)	92.21(5)
C(6)-Ru(1)-C(4)	68.08(6)
C(7)-Ru(1)-C(4)	80.33(6)
C(5)-Ru(1)-C(4)	38.25(6)
N(1)-Ru(1)-C(2)	126.60(6)
O(1)-Ru(1)-C(2)	155.95(5)
C(6)-Ru(1)-C(2)	68.91(6)
C(7)-Ru(1)-C(2)	37.54(6)
C(5)-Ru(1)-C(2)	82.28(6)
C(4)-Ru(1)-C(2)	68.22(6)
N(1)-Ru(1)-C(3)	164.61(6)
O(1)-Ru(1)-C(3)	118.13(5)
C(6)-Ru(1)-C(3)	80.45(6)
C(7)-Ru(1)-C(3)	67.71(6)
C(5)-Ru(1)-C(3)	68.54(6)
C(4)-Ru(1)-C(3)	37.04(6)
C(2)-Ru(1)-C(3)	38.05(6)
N(1)-Ru(1)-Cl(1)	85.41(4)
O(1)-Ru(1)-Cl(1)	85.52(3)
C(6)-Ru(1)-Cl(1)	156.07(5)
C(7)-Ru(1)-Cl(1)	117.86(5)
C(5)-Ru(1)-Cl(1)	157.00(4)
C(4)-Ru(1)-Cl(1)	119.11(5)

C(2)-Ru(1)-Cl(1)	91.90(5)
C(3)-Ru(1)-Cl(1)	93.26(5)
C(2)-C(7)-C(6)	120.82(15)
C(2)-C(7)-Ru(1)	72.14(9)
C(6)-C(7)-Ru(1)	70.23(9)
C(10)-C(8)-C(5)	113.54(14)
C(10)-C(8)-C(9)	110.71(16)
C(5)-C(8)-C(9)	111.53(14)
C(6)-C(5)-C(4)	117.74(15)
C(6)-C(5)-C(8)	123.60(15)
C(4)-C(5)-C(8)	118.44(14)
C(6)-C(5)-Ru(1)	70.30(9)
C(4)-C(5)-Ru(1)	71.59(9)
C(8)-C(5)-Ru(1)	124.62(11)
C(5)-C(6)-C(7)	120.97(15)
C(5)-C(6)-Ru(1)	71.58(9)
C(7)-C(6)-Ru(1)	71.29(9)
C(3)-C(4)-C(5)	121.29(15)
C(3)-C(4)-Ru(1)	71.81(9)
C(5)-C(4)-Ru(1)	70.16(9)
C(4)-C(3)-C(2)	120.85(15)
C(4)-C(3)-Ru(1)	71.15(9)
C(2)-C(3)-Ru(1)	70.72(9)
C(7)-C(2)-C(3)	118.27(15)
C(7)-C(2)-C(1)	121.97(16)
C(3)-C(2)-C(1)	119.68(16)
C(7)-C(2)-Ru(1)	70.32(9)
C(3)-C(2)-Ru(1)	71.23(9)
C(1)-C(2)-Ru(1)	127.24(12)
C(11)-N(1)-C(14)	107.08(13)
C(11)-N(1)-Ru(1)	138.62(11)
C(14)-N(1)-Ru(1)	114.24(10)
C(15)-O(1)-Ru(1)	115.49(9)
C(21)-C(16)-C(17)	118.79(14)
C(21)-C(16)-C(15)	120.40(14)
C(17)-C(16)-C(15)	120.76(13)

O(1)-C(15)-C(14)	118.27(14)
O(1)-C(15)-C(16)	119.58(13)
C(14)-C(15)-C(16)	122.13(13)
N(1)-C(14)-C(15)	114.76(13)
N(1)-C(14)-C(13)	109.13(13)
C(15)-C(14)-C(13)	136.08(15)
C(13)-C(12)-C(11)	107.05(14)
C(12)-C(13)-C(14)	106.28(14)
N(1)-C(11)-C(12)	110.45(14)
O(2)-C(17)-C(18)	123.87(14)
O(2)-C(17)-C(16)	116.23(14)
C(18)-C(17)-C(16)	119.85(14)
C(20)-C(21)-C(16)	121.29(15)
C(19)-C(18)-C(17)	119.89(15)
C(19)-C(20)-C(21)	119.16(15)
C(20)-C(19)-C(18)	120.98(15)
C(17)-O(2)-C(22)	117.49(13)

Symmetry transformations used to generate equivalent atoms:

Table 4. Anisotropic displacement parameters ($Å^2 x 10^3$) for 2. The anisotropic displacement factor exponent takes the form: $-2\pi^2[h^2 a^{*2}U^{11} + ... + 2 h k a^* b^* U^{12}]$

	U^{11}	U^{22}	U^{33}	U^{23}	U^{13}	U^{12}
Ru(1)	21(1)	20(1)	17(1)	0(1)	-1(1)	3(1)
Cl(1)	24(1)	32(1)	31(1)	1(1)	2(1)	-2(1)
C(7)	32(1)	21(1)	25(1)	-1(1)	-2(1)	4(1)
C(8)	24(1)	27(1)	28(1)	-4(1)	-3(1)	2(1)
C(5)	25(1)	23(1)	22(1)	-5(1)	-3(1)	0(1)
C(6)	29(1)	23(1)	24(1)	-2(1)	0(1)	-2(1)
C(4)	30(1)	25(1)	19(1)	-2(1)	-4(1)	1(1)
C(3)	34(1)	26(1)	20(1)	-3(1)	2(1)	2(1)
C(1)	36(1)	39(1)	38(1)	-5(1)	7(1)	11(1)
C(9)	26(1)	45(1)	37(1)	-8(1)	-5(1)	1(1)
C(10)	38(1)	58(1)	34(1)	1(1)	9(1)	14(1)
C(2)	32(1)	26(1)	24(1)	-5(1)	1(1)	6(1)
N(1)	19(1)	23(1)	20(1)	1(1)	0(1)	1(1)
O(1)	24(1)	24(1)	19(1)	-1(1)	-1(1)	4(1)
C(16)	20(1)	24(1)	20(1)	1(1)	4(1)	1(1)
C(15)	15(1)	24(1)	21(1)	-2(1)	1(1)	-1(1)
C(14)	19(1)	24(1)	20(1)	0(1)	1(1)	-1(1)
C(12)	24(1)	36(1)	20(1)	6(1)	0(1)	-2(1)
C(13)	22(1)	33(1)	20(1)	0(1)	2(1)	-3(1)
C(11)	18(1)	26(1)	26(1)	6(1)	0(1)	-1(1)
C(17)	21(1)	24(1)	20(1)	2(1)	3(1)	1(1)
C(21)	20(1)	28(1)	24(1)	-1(1)	3(1)	-2(1)
C(18)	25(1)	28(1)	26(1)	3(1)	5(1)	7(1)
C(20)	32(1)	27(1)	25(1)	-4(1)	6(1)	-4(1)
C(19)	35(1)	26(1)	25(1)	-1(1)	8(1)	6(1)
O(2)	19(1)	31(1)	33(1)	-5(1)	-6(1)	4(1)
C(22)	21(1)	36(1)	44(1)	2(1)	-8(1)	2(1)

Table 5. Hydrogen coordinates (x 10^4) and isotropic displacement parameters (Å^{2}x 10^3) for 2.

	x	y	z	U(eq)
H(7)	6135	99	1139	32
H(8)	2596	4124	645	33
H(6)	4023	840	1261	31
H(4)	3991	3719	-222	31
H(3)	6080	3004	-335	32
H(1A)	8070	433	687	56
H(1B)	8247	2022	396	56
H(1C)	7763	688	-71	56
H(9A)	1247	1596	104	56
H(9B)	1682	2918	-309	56
H(9C)	628	3184	151	56
H(10A)	1935	1589	1296	64
H(10B)	1333	3185	1344	64
H(10C)	2818	2888	1631	64
H(12)	6782	2873	3475	32
H(13)	5694	5293	3192	30
H(11)	6834	1520	2445	28
H(21)	6006	7663	2549	29
H(18)	1720	8898	1754	31
H(20)	5150	9862	2853	33
H(19)	3003	10453	2463	34
H(22A)	660	6770	1476	52
H(22B)	700	5862	832	52
H(22C)	984	7581	849	52

Table 1. Crystal data and structure refinement for 3.

Identification code	3	
Empirical formula	C53 H48 Cl N O4 P2 Ru	
Formula weight	961.38	
Temperature	150(2) K	
Wavelength	0.71073 Å	
Crystal system	Monoclinic	
Space group	$P2_1/n$	
Unit cell dimensions	a = 10.2279(5) Å	α= 90°.
	b = 17.5822(9) Å	β= 97.693(3)°.
	c = 25.9234(13) Å	γ = 90°.
Volume	4619.8(4) $Å^3$	
Z	4	
Density (calculated)	1.382 Mg/m^3	
Absorption coefficient	0.513 mm^{-1}	
F(000)	1824	
Crystal size	0.18 x 0.14 x 0.12 mm^3	
Theta range for data collection	1.40 to 25.00°.	
Index ranges	-12<=h<=12, -20<=k<=20, -30<=l<=30	
Reflections collected	49633	
Independent reflections	8132 [R(int) = 0.0979]	
Completeness to theta = 25.00°	100.0 %	
Absorption correction	Semi-empirical from equivalents	
Max. and min. transmission	0.9410 and 0.9133	
Refinement method	Full-matrix least-squares on F^2	
Data / restraints / parameters	8132 / 0 / 560	
Goodness-of-fit on F^2	0.880	
Final R indices [I>2sigma(I)]	R1 = 0.0591, wR2 = 0.1682	
R indices (all data)	R1 = 0.0879, wR2 = 0.1925	
Largest diff. peak and hole	0.811 and -0.925 e.$Å^{-3}$	

Table 2. Atomic coordinates (x 10^4) and equivalent isotropic displacement parameters ($Å^2$x 10^3) for 3. U(eq) is defined as one third of the trace of the orthogonalized U^{ij} tensor.

	x	y	z	U(eq)
Ru(1)	9642(1)	8351(1)	8102(1)	21(1)
Cl(1)	10933(1)	7559(1)	7598(1)	31(1)
P(1)	8581(1)	8980(1)	7333(1)	22(1)
P(2)	10657(1)	7770(1)	8889(1)	22(1)
N(1)	8886(4)	9200(3)	8508(2)	23(1)
O(1)	11228(4)	9153(2)	8189(1)	23(1)
O(2)	10725(5)	11242(3)	8022(2)	39(1)
C(50)	8197(6)	7721(4)	8093(2)	30(1)
O(4)	7293(5)	7361(3)	8114(2)	45(1)
C(1)	7073(5)	9442(3)	7472(2)	23(1)
C(2)	7040(6)	10200(3)	7645(2)	28(1)
C(3)	5876(6)	10510(4)	7770(2)	34(2)
C(4)	4752(6)	10076(4)	7740(2)	38(2)
C(5)	4771(6)	9324(4)	7596(2)	34(2)
O(3)	3010(9)	3975(5)	9738(4)	98(2)
C(6)	5928(5)	9010(4)	7455(2)	28(1)
C(7)	9546(6)	9704(4)	7052(2)	28(1)
C(8)	10891(6)	9571(4)	7063(2)	32(1)
C(9)	11686(7)	10092(4)	6848(3)	42(2)
C(10)	11142(7)	10744(4)	6614(3)	46(2)
C(11)	9814(7)	10873(4)	6591(3)	44(2)
C(12)	9008(6)	10364(4)	6805(3)	35(2)
C(13)	7917(6)	8398(3)	6773(2)	26(1)
C(14)	7209(7)	8741(4)	6338(3)	40(2)
C(15)	6614(7)	8308(5)	5928(3)	46(2)
C(16)	6733(7)	7531(4)	5944(3)	38(2)
C(17)	7428(7)	7184(4)	6363(3)	40(2)
C(18)	8036(6)	7621(4)	6779(3)	34(2)
C(19)	9830(6)	8008(4)	9449(2)	27(1)
C(20)	9869(7)	8757(4)	9634(2)	38(2)
C(21)	9170(8)	8969(4)	10032(3)	44(2)

C(22)	8393(7)	8443(5)	10247(3)	47(2)
C(23)	8342(7)	7712(5)	10065(3)	49(2)
C(24)	9041(6)	7497(4)	9668(3)	36(2)
C(25)	12399(6)	8020(3)	9081(2)	28(1)
C(26)	13243(6)	7960(4)	8709(3)	32(1)
C(27)	14568(6)	8100(4)	8833(3)	39(2)
C(28)	15076(7)	8306(4)	9333(3)	47(2)
C(29)	14256(7)	8377(5)	9709(3)	52(2)
C(30)	12911(7)	8231(4)	9586(2)	39(2)
C(31)	10725(5)	6736(3)	8937(2)	25(1)
C(32)	11360(7)	6403(4)	9390(3)	36(2)
C(33)	11363(7)	5629(4)	9456(3)	42(2)
C(34)	10704(7)	5163(4)	9067(3)	39(2)
C(35)	10088(6)	5476(4)	8619(2)	33(1)
C(36)	10102(6)	6268(3)	8549(2)	27(1)
C(37)	7806(6)	9357(4)	8729(2)	28(1)
C(38)	7910(6)	10087(4)	8951(2)	32(1)
C(39)	9087(6)	10388(3)	8859(2)	28(1)
C(40)	9704(5)	9838(3)	8583(2)	23(1)
C(41)	10944(5)	9766(3)	8416(2)	24(1)
C(42)	12001(5)	10349(3)	8532(2)	23(1)
C(43)	11874(6)	11088(4)	8334(2)	30(1)
C(44)	10566(8)	11992(4)	7807(3)	54(2)
C(45)	12907(7)	11600(4)	8452(3)	37(2)
C(46)	14034(7)	11383(4)	8774(3)	39(2)
C(47)	14165(6)	10660(4)	8972(3)	37(2)
C(48)	13147(6)	10139(4)	8848(2)	29(1)
C(54)	3834(16)	3942(8)	10199(6)	128(6)
C(53)	5115(16)	3795(10)	10099(10)	164(9)
C(51)	3683(19)	3869(11)	9317(6)	146(7)
C(52)	4960(20)	3557(15)	9523(10)	211(12)

Table 3. Bond lengths [Å] and angles [°] for 3.

Ru(1)-C(50)	1.845(7)
Ru(1)-N(1)	2.038(5)
Ru(1)-O(1)	2.139(4)
Ru(1)-P(2)	2.3908(15)
Ru(1)-P(1)	2.4058(15)
Ru(1)-Cl(1)	2.4185(15)
P(1)-C(1)	1.821(6)
P(1)-C(13)	1.832(6)
P(1)-C(7)	1.823(6)
P(2)-C(31)	1.824(6)
P(2)-C(19)	1.825(6)
P(2)-C(25)	1.838(6)
N(1)-C(37)	1.338(7)
N(1)-C(40)	1.398(7)
O(1)-C(41)	1.280(7)
O(2)-C(43)	1.362(7)
O(2)-C(44)	1.432(8)
C(50)-O(4)	1.128(8)
C(1)-C(6)	1.392(8)
C(1)-C(2)	1.408(8)
C(2)-C(3)	1.387(8)
C(2)-H(2)	0.9300
C(3)-C(4)	1.374(10)
C(3)-H(3)	0.9300
C(4)-C(5)	1.375(10)
C(4)-H(4)	0.9300
C(5)-C(6)	1.398(9)
C(5)-H(5)	0.9300
O(3)-C(54)	1.370(14)
O(3)-C(51)	1.377(18)
C(6)-H(6)	0.9300
C(7)-C(8)	1.391(8)
C(7)-C(12)	1.401(9)
C(8)-C(9)	1.390(9)

C(8)-H(8)	0.9300
C(9)-C(10)	1.378(10)
C(9)-H(9)	0.9300
C(10)-C(11)	1.370(10)
C(10)-H(10)	0.9300
C(11)-C(12)	1.382(9)
C(11)-H(11)	0.9300
C(12)-H(12)	0.9300
C(13)-C(18)	1.372(9)
C(13)-C(14)	1.393(9)
C(14)-C(15)	1.380(10)
C(14)-H(14)	0.9300
C(15)-C(16)	1.372(10)
C(15)-H(15)	0.9300
C(16)-C(17)	1.361(10)
C(16)-H(16)	0.9300
C(17)-C(18)	1.400(9)
C(17)-H(17)	0.9300
C(18)-H(18)	0.9300
C(19)-C(24)	1.379(9)
C(19)-C(20)	1.399(9)
C(20)-C(21)	1.383(10)
C(20)-H(20)	0.9300
C(21)-C(22)	1.386(11)
C(21)-H(21)	0.9300
C(22)-C(23)	1.368(11)
C(22)-H(22)	0.9300
C(23)-C(24)	1.382(10)
C(23)-H(23)	0.9300
C(24)-H(24)	0.9300
C(25)-C(26)	1.383(9)
C(25)-C(30)	1.393(9)
C(26)-C(27)	1.373(9)
C(26)-H(26)	0.9300
C(27)-C(28)	1.378(10)
C(27)-H(27)	0.9300

C(28)-C(29)	1.375(11)
C(28)-H(28)	0.9300
C(29)-C(30)	1.394(10)
C(29)-H(29)	0.9300
C(30)-H(30)	0.9300
C(31)-C(36)	1.385(8)
C(31)-C(32)	1.393(8)
C(32)-C(33)	1.371(10)
C(32)-H(32)	0.9300
C(33)-C(34)	1.402(9)
C(33)-H(33)	0.9300
C(34)-C(35)	1.360(9)
C(34)-H(34)	0.9300
C(35)-C(36)	1.406(9)
C(35)-H(35)	0.9300
C(36)-H(36)	0.9300
C(37)-C(38)	1.405(9)
C(37)-H(37)	0.9300
C(38)-C(39)	1.365(9)
C(38)-H(38)	0.9300
C(39)-C(40)	1.403(8)
C(39)-H(39)	0.9300
C(40)-C(41)	1.400(8)
C(41)-C(42)	1.490(8)
C(42)-C(48)	1.387(8)
C(42)-C(43)	1.396(9)
C(43)-C(45)	1.391(9)
C(44)-H(44A)	0.9600
C(44)-H(44B)	0.9600
C(44)-H(44C)	0.9600
C(45)-C(46)	1.383(10)
C(45)-H(45)	0.9300
C(46)-C(47)	1.371(10)
C(46)-H(46)	0.9300
C(47)-C(48)	1.392(9)
C(47)-H(47)	0.9300

C(48)-H(48)	0.9300
C(54)-C(53)	1.39(2)
C(54)-H(54A)	0.9700
C(54)-H(54B)	0.9700
C(53)-C(52)	1.54(3)
C(53)-H(53A)	0.9700
C(53)-H(53B)	0.9700
C(51)-C(52)	1.45(2)
C(51)-H(51A)	0.9700
C(51)-H(51B)	0.9700
C(52)-H(52A)	0.9700
C(52)-H(52B)	0.9700
C(50)-Ru(1)-N(1)	95.0(2)
C(50)-Ru(1)-O(1)	172.9(2)
N(1)-Ru(1)-O(1)	78.32(16)
C(50)-Ru(1)-P(2)	90.52(18)
N(1)-Ru(1)-P(2)	91.33(13)
O(1)-Ru(1)-P(2)	87.45(11)
C(50)-Ru(1)-P(1)	89.66(18)
N(1)-Ru(1)-P(1)	86.05(13)
O(1)-Ru(1)-P(1)	92.06(11)
P(2)-Ru(1)-P(1)	177.38(5)
C(50)-Ru(1)-Cl(1)	98.2(2)
N(1)-Ru(1)-Cl(1)	166.70(14)
O(1)-Ru(1)-Cl(1)	88.61(11)
P(2)-Ru(1)-Cl(1)	90.48(5)
P(1)-Ru(1)-Cl(1)	92.08(5)
C(1)-P(1)-C(13)	99.9(3)
C(1)-P(1)-C(7)	106.6(3)
C(13)-P(1)-C(7)	103.6(3)
C(1)-P(1)-Ru(1)	109.97(19)
C(13)-P(1)-Ru(1)	118.6(2)
C(7)-P(1)-Ru(1)	116.36(19)
C(31)-P(2)-C(19)	101.1(3)
C(31)-P(2)-C(25)	101.1(3)

C(19)-P(2)-C(25)	105.4(3)
C(31)-P(2)-Ru(1)	119.5(2)
C(19)-P(2)-Ru(1)	112.77(19)
C(25)-P(2)-Ru(1)	115.1(2)
C(37)-N(1)-C(40)	106.7(5)
C(37)-N(1)-Ru(1)	139.8(4)
C(40)-N(1)-Ru(1)	113.5(4)
C(41)-O(1)-Ru(1)	112.9(3)
C(43)-O(2)-C(44)	117.3(5)
O(4)-C(50)-Ru(1)	175.7(6)
C(6)-C(1)-C(2)	118.1(5)
C(6)-C(1)-P(1)	118.8(5)
C(2)-C(1)-P(1)	122.8(4)
C(3)-C(2)-C(1)	120.3(6)
C(3)-C(2)-H(2)	119.9
C(1)-C(2)-H(2)	119.9
C(4)-C(3)-C(2)	120.5(6)
C(4)-C(3)-H(3)	119.8
C(2)-C(3)-H(3)	119.8
C(3)-C(4)-C(5)	120.5(6)
C(3)-C(4)-H(4)	119.8
C(5)-C(4)-H(4)	119.8
C(4)-C(5)-C(6)	119.6(6)
C(4)-C(5)-H(5)	120.2
C(6)-C(5)-H(5)	120.2
C(54)-O(3)-C(51)	111.8(13)
C(1)-C(6)-C(5)	121.0(6)
C(1)-C(6)-H(6)	119.5
C(5)-C(6)-H(6)	119.5
C(8)-C(7)-C(12)	118.3(6)
C(8)-C(7)-P(1)	117.5(5)
C(12)-C(7)-P(1)	124.1(5)
C(7)-C(8)-C(9)	120.7(6)
C(7)-C(8)-H(8)	119.6
C(9)-C(8)-H(8)	119.6
C(10)-C(9)-C(8)	120.1(7)

C(10)-C(9)-H(9)	120.0
C(8)-C(9)-H(9)	120.0
C(11)-C(10)-C(9)	119.8(7)
C(11)-C(10)-H(10)	120.1
C(9)-C(10)-H(10)	120.1
C(10)-C(11)-C(12)	121.1(7)
C(10)-C(11)-H(11)	119.5
C(12)-C(11)-H(11)	119.5
C(11)-C(12)-C(7)	120.0(6)
C(11)-C(12)-H(12)	120.0
C(7)-C(12)-H(12)	120.0
C(18)-C(13)-C(14)	118.5(6)
C(18)-C(13)-P(1)	121.6(5)
C(14)-C(13)-P(1)	119.9(5)
C(15)-C(14)-C(13)	120.8(7)
C(15)-C(14)-H(14)	119.6
C(13)-C(14)-H(14)	119.6
C(16)-C(15)-C(14)	119.9(7)
C(16)-C(15)-H(15)	120.0
C(14)-C(15)-H(15)	120.0
C(17)-C(16)-C(15)	120.3(6)
C(17)-C(16)-H(16)	119.8
C(15)-C(16)-H(16)	119.8
C(16)-C(17)-C(18)	120.0(6)
C(16)-C(17)-H(17)	120.0
C(18)-C(17)-H(17)	120.0
C(13)-C(18)-C(17)	120.5(6)
C(13)-C(18)-H(18)	119.8
C(17)-C(18)-H(18)	119.8
C(24)-C(19)-C(20)	117.6(6)
C(24)-C(19)-P(2)	122.5(5)
C(20)-C(19)-P(2)	119.5(5)
C(21)-C(20)-C(19)	121.0(7)
C(21)-C(20)-H(20)	119.5
C(19)-C(20)-H(20)	119.5
C(22)-C(21)-C(20)	120.1(7)

C(22)-C(21)-H(21)	119.9
C(20)-C(21)-H(21)	119.9
C(23)-C(22)-C(21)	119.0(7)
C(23)-C(22)-H(22)	120.5
C(21)-C(22)-H(22)	120.5
C(22)-C(23)-C(24)	121.0(7)
C(22)-C(23)-H(23)	119.5
C(24)-C(23)-H(23)	119.5
C(19)-C(24)-C(23)	121.2(7)
C(19)-C(24)-H(24)	119.4
C(23)-C(24)-H(24)	119.4
C(26)-C(25)-C(30)	119.0(6)
C(26)-C(25)-P(2)	117.7(5)
C(30)-C(25)-P(2)	123.2(5)
C(27)-C(26)-C(25)	120.8(6)
C(27)-C(26)-H(26)	119.6
C(25)-C(26)-H(26)	119.6
C(26)-C(27)-C(28)	120.3(7)
C(26)-C(27)-H(27)	119.9
C(28)-C(27)-H(27)	119.9
C(29)-C(28)-C(27)	120.1(6)
C(29)-C(28)-H(28)	120.0
C(27)-C(28)-H(28)	120.0
C(28)-C(29)-C(30)	119.9(7)
C(28)-C(29)-H(29)	120.0
C(30)-C(29)-H(29)	120.0
C(29)-C(30)-C(25)	120.0(7)
C(29)-C(30)-H(30)	120.0
C(25)-C(30)-H(30)	120.0
C(36)-C(31)-C(32)	118.6(6)
C(36)-C(31)-P(2)	122.2(4)
C(32)-C(31)-P(2)	119.0(5)
C(33)-C(32)-C(31)	121.0(6)
C(33)-C(32)-H(32)	119.5
C(31)-C(32)-H(32)	119.5
C(32)-C(33)-C(34)	119.9(6)

C(32)-C(33)-H(33)	120.1
C(34)-C(33)-H(33)	120.1
C(35)-C(34)-C(33)	120.0(6)
C(35)-C(34)-H(34)	120.0
C(33)-C(34)-H(34)	120.0
C(34)-C(35)-C(36)	119.9(6)
C(34)-C(35)-H(35)	120.0
C(36)-C(35)-H(35)	120.0
C(31)-C(36)-C(35)	120.5(6)
C(31)-C(36)-H(36)	119.8
C(35)-C(36)-H(36)	119.8
N(1)-C(37)-C(38)	110.1(5)
N(1)-C(37)-H(37)	125.0
C(38)-C(37)-H(37)	125.0
C(39)-C(38)-C(37)	107.6(5)
C(39)-C(38)-H(38)	126.2
C(37)-C(38)-H(38)	126.2
C(38)-C(39)-C(40)	106.8(5)
C(38)-C(39)-H(39)	126.6
C(40)-C(39)-H(39)	126.6
N(1)-C(40)-C(39)	108.8(5)
N(1)-C(40)-C(41)	115.8(5)
C(39)-C(40)-C(41)	135.1(6)
O(1)-C(41)-C(40)	119.3(5)
O(1)-C(41)-C(42)	117.9(5)
C(40)-C(41)-C(42)	122.6(5)
C(48)-C(42)-C(43)	119.6(5)
C(48)-C(42)-C(41)	118.0(5)
C(43)-C(42)-C(41)	122.4(5)
O(2)-C(43)-C(45)	124.8(6)
O(2)-C(43)-C(42)	115.7(5)
C(45)-C(43)-C(42)	119.5(6)
O(2)-C(44)-H(44A)	109.5
O(2)-C(44)-H(44B)	109.5
H(44A)-C(44)-H(44B)	109.5
O(2)-C(44)-H(44C)	109.5

H(44A)-C(44)-H(44C)	109.5
H(44B)-C(44)-H(44C)	109.5
C(43)-C(45)-C(46)	120.0(6)
C(43)-C(45)-H(45)	120.0
C(46)-C(45)-H(45)	120.0
C(47)-C(46)-C(45)	120.9(6)
C(47)-C(46)-H(46)	119.5
C(45)-C(46)-H(46)	119.5
C(46)-C(47)-C(48)	119.4(6)
C(46)-C(47)-H(47)	120.3
C(48)-C(47)-H(47)	120.3
C(47)-C(48)-C(42)	120.5(6)
C(47)-C(48)-H(48)	119.7
C(42)-C(48)-H(48)	119.7
O(3)-C(54)-C(53)	109.2(16)
O(3)-C(54)-H(54A)	109.8
C(53)-C(54)-H(54A)	109.8
O(3)-C(54)-H(54B)	109.8
C(53)-C(54)-H(54B)	109.8
H(54A)-C(54)-H(54B)	108.3
C(54)-C(53)-C(52)	104.8(15)
C(54)-C(53)-H(53A)	110.8
C(52)-C(53)-H(53A)	110.8
C(54)-C(53)-H(53B)	110.8
C(52)-C(53)-H(53B)	110.8
H(53A)-C(53)-H(53B)	108.9
O(3)-C(51)-C(52)	106.3(14)
O(3)-C(51)-H(51A)	110.5
C(52)-C(51)-H(51A)	110.5
O(3)-C(51)-H(51B)	110.5
C(52)-C(51)-H(51B)	110.5
H(51A)-C(51)-H(51B)	108.7
C(51)-C(52)-C(53)	102.9(14)
C(51)-C(52)-H(52A)	111.2
C(53)-C(52)-H(52A)	111.2
C(51)-C(52)-H(52B)	111.2

C(53)-C(52)-H(52B)	111.2
H(52A)-C(52)-H(52B)	109.1

Symmetry transformations used to generate equivalent atoms:

Table 4. Anisotropic displacement parameters ($Å^2$x 10^3) for 3. The anisotropic displacement factor exponent takes the form: $-2\pi^2$[h^2 $a^{*2}U^{11}$ + ... + 2 h k a* b* U^{12}]

	U^{11}	U^{22}	U^{33}	U^{23}	U^{13}	U^{12}
Ru(1)	14(1)	20(1)	28(1)	-1(1)	1(1)	0(1)
Cl(1)	24(1)	32(1)	36(1)	-5(1)	3(1)	4(1)
P(1)	16(1)	20(1)	29(1)	0(1)	2(1)	0(1)
P(2)	17(1)	21(1)	27(1)	-1(1)	2(1)	0(1)
N(1)	15(2)	23(3)	30(2)	1(2)	-1(2)	-6(2)
O(1)	18(2)	21(2)	28(2)	-2(2)	0(2)	-1(2)
O(2)	39(3)	30(3)	44(3)	8(2)	-5(2)	-2(2)
C(50)	33(4)	22(3)	33(3)	4(3)	-4(3)	11(3)
O(4)	27(3)	43(3)	62(3)	10(2)	-3(2)	-4(2)
C(1)	16(3)	23(3)	30(3)	3(2)	2(2)	1(2)
C(2)	23(3)	24(3)	35(3)	0(3)	0(2)	3(2)
C(3)	30(3)	38(4)	33(3)	-8(3)	6(3)	9(3)
C(4)	22(3)	62(5)	31(3)	2(3)	4(3)	18(3)
C(5)	25(3)	39(4)	39(3)	5(3)	2(3)	-3(3)
O(3)	104(6)	83(6)	104(6)	28(5)	4(5)	21(5)
C(6)	18(3)	26(3)	39(3)	1(3)	1(2)	-1(2)
C(7)	22(3)	31(3)	29(3)	0(3)	1(2)	-1(3)
C(8)	20(3)	42(4)	34(3)	2(3)	4(2)	-2(3)
C(9)	26(3)	59(5)	41(4)	4(3)	8(3)	-7(3)
C(10)	47(4)	46(5)	45(4)	8(3)	11(3)	-10(4)
C(11)	46(4)	36(4)	52(4)	13(3)	13(3)	0(3)
C(12)	23(3)	32(4)	50(4)	8(3)	9(3)	4(3)
C(13)	22(3)	25(3)	32(3)	-6(3)	3(2)	-1(2)
C(14)	50(4)	31(4)	39(4)	-4(3)	-1(3)	5(3)
C(15)	48(4)	55(5)	32(4)	-4(3)	-7(3)	9(4)
C(16)	36(4)	43(4)	35(3)	-16(3)	2(3)	-4(3)
C(17)	38(4)	28(4)	53(4)	-11(3)	-1(3)	2(3)
C(18)	25(3)	27(4)	47(4)	-4(3)	-3(3)	4(3)
C(19)	20(3)	28(3)	32(3)	2(3)	-1(2)	5(3)
C(20)	49(4)	35(4)	28(3)	1(3)	3(3)	5(3)
C(21)	57(5)	41(4)	31(3)	-6(3)	-4(3)	16(4)

C(22)	29(4)	70(6)	41(4)	-8(4)	8(3)	10(4)
C(23)	28(4)	76(6)	46(4)	-5(4)	15(3)	-13(4)
C(24)	24(3)	38(4)	45(4)	-9(3)	2(3)	-6(3)
C(25)	21(3)	24(3)	37(3)	6(3)	-3(3)	-2(3)
C(26)	24(3)	33(4)	38(3)	0(3)	-2(3)	-3(3)
C(27)	23(3)	42(4)	52(4)	5(3)	3(3)	-2(3)
C(28)	24(3)	56(5)	58(5)	10(4)	-5(3)	-10(3)
C(29)	36(4)	74(6)	41(4)	-1(4)	-11(3)	-18(4)
C(30)	34(4)	50(5)	30(3)	-1(3)	-3(3)	-8(3)
C(31)	17(3)	25(3)	35(3)	1(2)	6(2)	4(2)
C(32)	39(4)	27(3)	38(4)	0(3)	-9(3)	3(3)
C(33)	43(4)	35(4)	43(4)	8(3)	-9(3)	6(3)
C(34)	44(4)	14(3)	56(4)	2(3)	-3(3)	2(3)
C(35)	27(3)	33(4)	39(3)	-6(3)	2(3)	-8(3)
C(36)	24(3)	23(3)	35(3)	-1(3)	1(2)	4(2)
C(37)	18(3)	35(4)	31(3)	1(3)	6(2)	3(3)
C(38)	22(3)	34(4)	39(3)	-4(3)	5(3)	10(3)
C(39)	26(3)	21(3)	35(3)	0(3)	1(2)	4(3)
C(40)	16(3)	24(3)	30(3)	1(2)	2(2)	-1(2)
C(41)	18(3)	24(3)	29(3)	1(2)	-2(2)	1(2)
C(42)	15(3)	21(3)	35(3)	-6(2)	5(2)	-4(2)
C(43)	25(3)	29(3)	36(3)	-4(3)	5(3)	-2(3)
C(44)	57(5)	33(4)	68(5)	18(4)	-5(4)	5(4)
C(45)	41(4)	26(4)	46(4)	-2(3)	13(3)	-11(3)
C(46)	29(4)	39(4)	52(4)	-15(3)	13(3)	-13(3)
C(47)	19(3)	53(5)	40(4)	-12(3)	2(3)	-3(3)
C(48)	20(3)	31(4)	36(3)	-6(3)	1(2)	-2(3)
C(54)	134(13)	77(9)	154(13)	8(9)	-53(11)	-30(9)
C(53)	70(10)	107(12)	310(30)	32(15)	-12(14)	22(9)
C(51)	168(17)	152(16)	125(13)	65(12)	46(12)	19(13)
C(52)	180(20)	240(30)	240(30)	10(20)	140(20)	80(20)

Table 5. Hydrogen coordinates (x 10^4) and isotropic displacement parameters ($Å^2$x 10^3) for 3.

	x	y	z	U(eq)
H(2)	7802	10494	7675	33
H(3)	5856	11015	7875	40
H(4)	3972	10292	7818	45
H(5)	4018	9026	7592	41
H(6)	5932	8505	7349	34
H(8)	11262	9129	7216	38
H(9)	12585	10001	6861	50
H(10)	11674	11095	6472	55
H(11)	9450	11310	6429	53
H(12)	8108	10459	6784	42
H(14)	7138	9268	6323	49
H(15)	6133	8543	5642	55
H(16)	6336	7240	5667	46
H(17)	7500	6657	6373	48
H(18)	8524	7382	7062	41
H(20)	10374	9117	9487	45
H(21)	9221	9467	10155	53
H(22)	7913	8584	10512	56
H(23)	7829	7354	10210	59
H(24)	8979	7000	9546	43
H(26)	12908	7824	8370	39
H(27)	15125	8054	8579	47
H(28)	15976	8399	9415	56
H(29)	14598	8521	10045	62
H(30)	12355	8275	9841	46
H(32)	11788	6710	9652	43
H(33)	11802	5415	9759	50
H(34)	10687	4639	9114	47
H(35)	9659	5165	8359	40
H(36)	9691	6480	8241	33

H(37)	7092	9031	8734	33
H(38)	7289	10322	9129	38
H(39)	9416	10867	8960	34
H(44A)	11268	12096	7606	81
H(44B)	10588	12356	8084	81
H(44C)	9735	12025	7587	81
H(45)	12840	12089	8314	44
H(46)	14712	11732	8857	47
H(47)	14927	10519	9187	45
H(48)	13235	9646	8978	35
H(54A)	3547	3543	10417	154
H(54B)	3811	4422	10383	154
H(53A)	5511	3389	10321	197
H(53B)	5661	4245	10157	197
H(51A)	3788	4349	9143	175
H(51B)	3208	3520	9070	175
H(52A)	5657	3772	9348	254
H(52B)	4971	3008	9488	254

Table 1 Identification code	4	
Empirical formula	C23 H24 N2 O3 Ru	
Formula weight	477.51	
Temperature	150(2) K	
Wavelength	0.71073 Å	
Crystal system	Monoclinic	
Space group	$P2_1/c$	
Unit cell dimensions	a = 10.157(5) Å	α= 90°.
	b = 15.836(7) Å	β= 105.44(3)°.
	c = 14.019(8) Å	γ = 90°.
Volume	2173.5(19) $Å^3$	
Z	4	
Density (calculated)	1.465 Mg/m^3	
Absorption coefficient	0.748 mm^{-1}	
F(000)	980	
Crystal size	0.180 x 0.140 x 0.120 mm^3	
Theta range for data collection	1.981 to 28.281°.	
Index ranges	-13<=h<=13, -21<=k<=21, -18<=l<=13	
Reflections collected	26439	
Independent reflections	5380 [R(int) = 0.0893]	
Completeness to theta = 25.242°	99.9 %	
Refinement method	Full-matrix least-squares on F^2	
Data / restraints / parameters	5380 / 0 / 266	
Goodness-of-fit on F^2	0.968	
Final R indices [I>2sigma(I)]	R1 = 0.0568, wR2 = 0.1449	
R indices (all data)	R1 = 0.0890, wR2 = 0.1724	
Extinction coefficient	n/a	
Largest diff. peak and hole	1.071 and -2.014 e.$Å^{-3}$	

Table 2 (x 10^4) and equivalent isotropic displacement parameters ($Å^2$x 10^3) for 4. U(eq) is defined as one third of the trace of the orthogonalized U^{ij} tensor.

	x	y	z	U(eq)
Ru(1)	-242(1)	8644(1)	1100(1)	32(1)
O(1)	-2459(4)	8030(3)	-1937(3)	69(1)
O(2)	1228(3)	8528(2)	278(2)	38(1)
O(3)	3995(4)	6830(3)	711(3)	59(1)
N(1)	-1625(5)	8455(3)	-262(3)	49(1)
C(23)	-2004(5)	8230(3)	-1073(4)	41(1)
N(2)	54(4)	7361(2)	1070(3)	35(1)
C(18)	-562(6)	8665(3)	2583(4)	40(1)
C(16)	816(5)	8879(3)	2655(4)	41(1)
C(14)	1133(5)	9527(3)	2076(4)	39(1)
C(15)	56(5)	9984(2)	1434(4)	37(1)
C(17)	-1298(4)	9780(2)	1352(4)	39(1)
C(19)	-1647(5)	9111(3)	1941(4)	42(1)
C(20)	-3115(6)	8894(3)	1919(5)	57(2)
C(22)	-3445(7)	9338(4)	2821(6)	85(2)
C(21)	-4146(6)	9132(4)	964(5)	82(2)
C(13)	2600(5)	9704(3)	2079(5)	59(2)
C(5)	1580(4)	7776(2)	124(3)	33(1)
C(6)	2545(5)	7677(2)	-487(3)	34(1)
C(11)	3748(5)	7201(3)	-202(4)	43(1)
C(12)	5164(8)	6315(4)	1035(5)	79(2)
C(10)	4610(6)	7150(3)	-828(4)	55(1)
C(9)	4314(6)	7568(3)	-1725(4)	56(1)
C(8)	3120(6)	8041(3)	-2025(4)	49(1)
C(7)	2264(5)	8099(3)	-1403(4)	39(1)
C(4)	950(5)	7115(2)	514(4)	37(1)
C(1)	-473(5)	6665(3)	1352(4)	44(1)
C(2)	80(6)	5959(3)	995(4)	52(1)
C(3)	961(6)	6234(3)	465(4)	47(1)

Table 3 4.

Ru(1)-N(2)	2.057(3)
Ru(1)-N(1)	2.070(5)
Ru(1)-O(2)	2.124(3)
Ru(1)-C(17)	2.171(4)
Ru(1)-C(15)	2.177(4)
Ru(1)-C(14)	2.181(4)
Ru(1)-C(18)	2.188(5)
Ru(1)-C(16)	2.189(5)
Ru(1)-C(19)	2.206(5)
O(1)-C(23)	1.218(6)
O(2)-C(5)	1.278(5)
O(3)-C(11)	1.370(6)
O(3)-C(12)	1.412(7)
N(1)-C(23)	1.155(6)
N(2)-C(1)	1.330(6)
N(2)-C(4)	1.401(6)
C(18)-C(19)	1.412(7)
C(18)-C(16)	1.417(8)
C(18)-H(18)	0.9300
C(16)-C(14)	1.398(7)
C(16)-H(16)	0.9300
C(14)-C(15)	1.417(6)
C(14)-C(13)	1.515(6)
C(15)-C(17)	1.388(6)
C(15)-H(15)	0.9300
C(17)-C(19)	1.445(6)
C(17)-H(17)	0.9300
C(19)-C(20)	1.523(7)
C(20)-C(21)	1.512(8)
C(20)-C(22)	1.560(8)
C(20)-H(20)	0.9800
C(22)-H(22A)	0.9600
C(22)-H(22B)	0.9600
C(22)-H(22C)	0.9600

C(21)-H(21A)	0.9600
C(21)-H(21B)	0.9600
C(21)-H(21C)	0.9600
C(13)-H(13A)	0.9600
C(13)-H(13B)	0.9600
C(13)-H(13C)	0.9600
C(5)-C(4)	1.411(6)
C(5)-C(6)	1.472(6)
C(6)-C(11)	1.401(6)
C(6)-C(7)	1.408(6)
C(11)-C(10)	1.397(7)
C(12)-H(12A)	0.9600
C(12)-H(12B)	0.9600
C(12)-H(12C)	0.9600
C(10)-C(9)	1.382(8)
C(10)-H(10)	0.9300
C(9)-C(8)	1.391(7)
C(9)-H(9)	0.9300
C(8)-C(7)	1.389(7)
C(8)-H(8)	0.9300
C(7)-H(7)	0.9300
C(4)-C(3)	1.398(6)
C(1)-C(2)	1.401(8)
C(1)-H(1)	0.9300
C(2)-C(3)	1.377(8)
C(2)-H(2)	0.9300
C(3)-H(3)	0.9300
N(2)-Ru(1)-N(1)	84.53(16)
N(2)-Ru(1)-O(2)	77.03(13)
N(1)-Ru(1)-O(2)	83.61(16)
N(2)-Ru(1)-C(17)	154.61(16)
N(1)-Ru(1)-C(17)	91.52(17)
O(2)-Ru(1)-C(17)	127.54(15)
N(2)-Ru(1)-C(15)	162.79(18)
N(1)-Ru(1)-C(15)	111.15(17)

O(2)-Ru(1)-C(15)	97.09(14)
C(17)-Ru(1)-C(15)	37.22(16)
N(2)-Ru(1)-C(14)	125.00(17)
N(1)-Ru(1)-C(14)	146.67(17)
O(2)-Ru(1)-C(14)	88.32(15)
C(17)-Ru(1)-C(14)	68.21(17)
C(15)-Ru(1)-C(14)	37.95(17)
N(2)-Ru(1)-C(18)	95.40(15)
N(1)-Ru(1)-C(18)	130.0(2)
O(2)-Ru(1)-C(18)	145.20(18)
C(17)-Ru(1)-C(18)	68.17(18)
C(15)-Ru(1)-C(18)	80.13(17)
C(14)-Ru(1)-C(18)	68.10(18)
N(2)-Ru(1)-C(16)	98.77(17)
N(1)-Ru(1)-C(16)	167.36(18)
O(2)-Ru(1)-C(16)	109.01(17)
C(17)-Ru(1)-C(16)	80.25(18)
C(15)-Ru(1)-C(16)	67.53(18)
C(14)-Ru(1)-C(16)	37.32(18)
C(18)-Ru(1)-C(16)	37.8(2)
N(2)-Ru(1)-C(19)	117.43(16)
N(1)-Ru(1)-C(19)	99.47(19)
O(2)-Ru(1)-C(19)	165.35(13)
C(17)-Ru(1)-C(19)	38.54(17)
C(15)-Ru(1)-C(19)	68.39(16)
C(14)-Ru(1)-C(19)	81.26(18)
C(18)-Ru(1)-C(19)	37.49(18)
C(16)-Ru(1)-C(19)	68.13(19)
C(5)-O(2)-Ru(1)	116.2(3)
C(11)-O(3)-C(12)	118.5(5)
C(23)-N(1)-Ru(1)	156.9(4)
N(1)-C(23)-O(1)	176.3(6)
C(1)-N(2)-C(4)	107.9(4)
C(1)-N(2)-Ru(1)	137.8(3)
C(4)-N(2)-Ru(1)	113.9(3)
C(19)-C(18)-C(16)	121.0(4)

C(19)-C(18)-Ru(1)	71.9(3)
C(16)-C(18)-Ru(1)	71.2(3)
C(19)-C(18)-H(18)	119.5
C(16)-C(18)-H(18)	119.5
Ru(1)-C(18)-H(18)	130.0
C(14)-C(16)-C(18)	120.6(4)
C(14)-C(16)-Ru(1)	71.0(3)
C(18)-C(16)-Ru(1)	71.0(3)
C(14)-C(16)-H(16)	119.7
C(18)-C(16)-H(16)	119.7
Ru(1)-C(16)-H(16)	131.0
C(16)-C(14)-C(15)	119.1(4)
C(16)-C(14)-C(13)	120.9(4)
C(15)-C(14)-C(13)	119.9(4)
C(16)-C(14)-Ru(1)	71.7(3)
C(15)-C(14)-Ru(1)	70.9(3)
C(13)-C(14)-Ru(1)	125.8(3)
C(17)-C(15)-C(14)	120.9(4)
C(17)-C(15)-Ru(1)	71.1(2)
C(14)-C(15)-Ru(1)	71.2(2)
C(17)-C(15)-H(15)	119.5
C(14)-C(15)-H(15)	119.5
Ru(1)-C(15)-H(15)	130.9
C(15)-C(17)-C(19)	120.8(4)
C(15)-C(17)-Ru(1)	71.6(2)
C(19)-C(17)-Ru(1)	72.0(2)
C(15)-C(17)-H(17)	119.6
C(19)-C(17)-H(17)	119.6
Ru(1)-C(17)-H(17)	129.2
C(18)-C(19)-C(17)	117.5(4)
C(18)-C(19)-C(20)	119.6(5)
C(17)-C(19)-C(20)	122.8(5)
C(18)-C(19)-Ru(1)	70.6(3)
C(17)-C(19)-Ru(1)	69.4(2)
C(20)-C(19)-Ru(1)	133.1(4)
C(21)-C(20)-C(19)	113.9(5)

C(21)-C(20)-C(22)	111.0(5)
C(19)-C(20)-C(22)	107.6(5)
C(21)-C(20)-H(20)	108.1
C(19)-C(20)-H(20)	108.1
C(22)-C(20)-H(20)	108.1
C(20)-C(22)-H(22A)	109.5
C(20)-C(22)-H(22B)	109.5
H(22A)-C(22)-H(22B)	109.5
C(20)-C(22)-H(22C)	109.5
H(22A)-C(22)-H(22C)	109.5
H(22B)-C(22)-H(22C)	109.5
C(20)-C(21)-H(21A)	109.5
C(20)-C(21)-H(21B)	109.5
H(21A)-C(21)-H(21B)	109.5
C(20)-C(21)-H(21C)	109.5
H(21A)-C(21)-H(21C)	109.5
H(21B)-C(21)-H(21C)	109.5
C(14)-C(13)-H(13A)	109.5
C(14)-C(13)-H(13B)	109.5
H(13A)-C(13)-H(13B)	109.5
C(14)-C(13)-H(13C)	109.5
H(13A)-C(13)-H(13C)	109.5
H(13B)-C(13)-H(13C)	109.5
O(2)-C(5)-C(4)	116.7(4)
O(2)-C(5)-C(6)	117.2(4)
C(4)-C(5)-C(6)	126.0(4)
C(11)-C(6)-C(7)	117.7(4)
C(11)-C(6)-C(5)	123.9(4)
C(7)-C(6)-C(5)	118.4(4)
O(3)-C(11)-C(10)	125.0(4)
O(3)-C(11)-C(6)	115.2(4)
C(10)-C(11)-C(6)	119.8(5)
O(3)-C(12)-H(12A)	109.5
O(3)-C(12)-H(12B)	109.5
H(12A)-C(12)-H(12B)	109.5
O(3)-C(12)-H(12C)	109.5

H(12A)-C(12)-H(12C)	109.5
H(12B)-C(12)-H(12C)	109.5
C(9)-C(10)-C(11)	121.5(5)
C(9)-C(10)-H(10)	119.3
C(11)-C(10)-H(10)	119.3
C(10)-C(9)-C(8)	119.6(5)
C(10)-C(9)-H(9)	120.2
C(8)-C(9)-H(9)	120.2
C(7)-C(8)-C(9)	119.1(5)
C(7)-C(8)-H(8)	120.4
C(9)-C(8)-H(8)	120.4
C(8)-C(7)-C(6)	122.1(4)
C(8)-C(7)-H(7)	118.9
C(6)-C(7)-H(7)	118.9
C(3)-C(4)-N(2)	108.7(4)
C(3)-C(4)-C(5)	135.3(5)
N(2)-C(4)-C(5)	116.0(4)
N(2)-C(1)-C(2)	108.9(5)
N(2)-C(1)-H(1)	125.5
C(2)-C(1)-H(1)	125.5
C(3)-C(2)-C(1)	108.7(4)
C(3)-C(2)-H(2)	125.6
C(1)-C(2)-H(2)	125.6
C(2)-C(3)-C(4)	105.8(5)
C(2)-C(3)-H(3)	127.1
C(4)-C(3)-H(3)	127.1

Symmetry transformations used to generate equivalent atoms:

Table 4 (Å^{2}x 10^3) for 4. The anisotropic displacement factor exponent takes the form: $-2\pi^2$[h^2 $a^{*2}U^{11}$ + ... + 2 h k a* b* U^{12}]

	U^{11}	U^{22}	U^{33}	U^{23}	U^{13}	U^{12}
Ru(1)	36(1)	22(1)	39(1)	1(1)	11(1)	2(1)
O(1)	70(3)	75(3)	58(3)	-23(2)	11(2)	-4(2)
O(2)	47(2)	24(1)	46(2)	0(1)	18(2)	2(1)
O(3)	52(2)	75(3)	49(2)	13(2)	12(2)	27(2)
N(1)	51(3)	45(2)	48(3)	-3(2)	8(2)	5(2)
C(23)	34(3)	34(2)	54(3)	-3(2)	11(2)	0(2)
N(2)	43(2)	20(2)	44(2)	2(2)	12(2)	3(1)
C(18)	62(3)	27(2)	37(3)	-2(2)	21(2)	-1(2)
C(16)	48(3)	36(2)	35(3)	-6(2)	7(2)	7(2)
C(14)	40(3)	31(2)	47(3)	-8(2)	11(2)	-2(2)
C(15)	45(3)	21(2)	46(3)	-3(2)	15(2)	1(2)
C(17)	40(3)	24(2)	54(3)	-1(2)	14(2)	7(2)
C(19)	47(3)	30(2)	55(3)	-7(2)	26(2)	0(2)
C(20)	47(3)	44(3)	88(5)	-2(3)	33(3)	-1(2)
C(22)	76(5)	87(5)	113(6)	-8(4)	62(4)	-9(4)
C(21)	46(3)	89(5)	107(6)	-12(4)	14(4)	-5(3)
C(13)	39(3)	49(3)	82(4)	-6(3)	2(3)	-6(2)
C(5)	32(2)	28(2)	36(2)	-2(2)	2(2)	3(2)
C(6)	38(2)	26(2)	40(3)	-7(2)	12(2)	-1(2)
C(11)	45(3)	42(2)	45(3)	-3(2)	15(2)	11(2)
C(12)	80(4)	85(5)	66(5)	12(3)	8(4)	44(4)
C(10)	53(3)	57(3)	57(4)	-1(3)	19(3)	15(3)
C(9)	57(3)	56(3)	63(4)	-8(3)	31(3)	-1(3)
C(8)	67(3)	36(2)	48(3)	3(2)	22(3)	-3(2)
C(7)	47(3)	27(2)	43(3)	-1(2)	13(2)	2(2)
C(4)	45(3)	24(2)	43(3)	-3(2)	12(2)	3(2)
C(1)	55(3)	29(2)	53(3)	0(2)	20(3)	-8(2)
C(2)	73(4)	24(2)	62(4)	0(2)	23(3)	-8(2)
C(3)	65(4)	27(2)	51(3)	-6(2)	16(3)	4(2)

Table 5 displacement parameters (Å^{2}x 10 ^{3}) for 4.

	x	y	z	U(eq)
H(18)	-755	8226	2964	49
H(16)	1516	8585	3091	49
H(15)	260	10428	1062	44
H(17)	-1990	10078	913	47
H(20)	-3171	8282	2009	69
H(22A)	-3320	9937	2778	128
H(22B)	-2845	9128	3423	128
H(22C)	-4375	9224	2817	128
H(21A)	-3916	8856	419	123
H(21B)	-4135	9733	874	123
H(21C)	-5041	8958	991	123
H(13A)	3163	9233	2365	89
H(13B)	2913	10203	2461	89
H(13C)	2653	9788	1412	89
H(12A)	5968	6644	1066	119
H(12B)	5115	5856	580	119
H(12C)	5203	6095	1681	119
H(10)	5402	6827	-636	66
H(9)	4909	7534	-2126	67
H(8)	2898	8314	-2634	59
H(7)	1480	8429	-1598	47
H(1)	-1105	6649	1725	53
H(2)	-116	5399	1099	63
H(3)	1461	5901	140	57

Table 1. Crystal data and structure refinement for 5.

Identification code	5	
Empirical formula	C22 H24 N4 O2 Ru	
Formula weight	477.52	
Temperature	150 K	
Wavelength	0.71073 Å	
Crystal system	Monoclinic	
Space group	P2(1)/c	
Unit cell dimensions	a = 17.3849(14) Å	α= 90°.
	b = 9.2423(7) Å	β= 112.379(4)°.
	c = 13.8157(11) Å	γ = 90°.
Volume	2052.7(3) $Å^3$	
Z	4	
Density (calculated)	1.545 Mg/m^3	
Absorption coefficient	0.787 mm^{-1}	
F(000)	976.0	
Crystal size	0.18 x 0.14 x 0.12 mm^3	
Theta range for data collection	2.54 to 28.74°.	
Index ranges	-19<=h<=23, -12<=k<=12, -18<=l<=18	
Reflections collected	18124	
Independent reflections	5243 [R(int) = 0.0298]	
Completeness to theta = 28.74°	98.2 %	
Absorption correction	Semi-empirical from equivalents	
Max. and min. transmission	0.9196 and 0.8828	
Refinement method	Full-matrix least-squares on F^2	
Data / restraints / parameters	5243 / 0 / 266	
Goodness-of-fit on F^2	1.067	
Final R indices [I>2sigma(I)]	R1 = 0.0306, wR2 = 0.0783	
R indices (all data)	R1 = 0.0356, wR2 = 0.0810	
Largest diff. peak and hole	0.870 and -0.699 e.$Å^{-3}$	

Table 2. Atomic coordinates (x 10^4) and equivalent isotropic displacement parameters (Å^{2}x 10^3) for 5. U(eq) is defined as one third of the trace of the orthogonalized U^{ij} tensor.

	x	y	z	U(eq)
Ru(1)	6689(1)	5397(1)	6571(1)	19(1)
O(1)	7318(1)	6250(2)	5659(1)	24(1)
O(2)	8546(1)	8655(2)	5937(2)	36(1)
N(1)	6348(1)	3654(2)	5506(2)	29(1)
N(2)	6820(1)	3212(2)	5126(2)	26(1)
N(3)	7246(2)	2740(3)	4733(2)	38(1)
N(4)	7841(1)	4419(2)	7204(2)	20(1)
C(1)	5652(1)	6952(2)	6162(2)	24(1)
C(2)	6391(2)	7600(3)	6879(2)	27(1)
C(3)	6832(2)	6891(3)	7844(2)	32(1)
C(4)	6559(2)	5527(3)	8049(2)	35(1)
C(5)	5835(2)	4870(3)	7345(2)	32(1)
C(6)	5546(2)	3417(3)	7555(3)	57(1)
C(7)	5386(2)	5614(2)	6395(2)	27(1)
C(8)	6730(2)	9004(3)	6631(3)	40(1)
C(9)	6542(3)	10221(3)	7269(4)	63(1)
C(10)	6401(3)	9384(3)	5480(3)	55(1)
C(11)	8390(1)	4835(2)	6742(2)	21(1)
C(12)	9150(2)	4099(3)	7234(2)	26(1)
C(13)	9048(2)	3225(3)	7990(2)	28(1)
C(14)	8233(2)	3452(2)	7945(2)	24(1)
C(15)	8064(1)	5806(2)	5909(2)	22(1)
C(16)	8528(1)	6327(2)	5262(2)	24(1)
C(17)	8711(2)	5367(3)	4610(2)	32(1)
C(18)	9053(2)	5854(3)	3907(2)	39(1)
C(19)	9222(2)	7313(3)	3880(2)	37(1)
C(20)	9064(2)	8288(3)	4537(2)	30(1)
C(21)	8720(2)	7803(3)	5243(2)	25(1)
C(22)	8707(2)	10173(3)	5920(3)	39(1)

Table 3. Bond lengths [Å] and angles [°] for 5.

Ru(1)-N(4)	2.0640(19)
Ru(1)-N(1)	2.109(2)
Ru(1)-O(1)	2.1114(15)
Ru(1)-C(4)	2.142(2)
Ru(1)-C(3)	2.174(2)
Ru(1)-C(2)	2.182(2)
Ru(1)-C(5)	2.189(2)
Ru(1)-C(7)	2.193(2)
Ru(1)-C(1)	2.205(2)
O(1)-C(15)	1.276(3)
O(2)-C(21)	1.361(3)
O(2)-C(22)	1.433(3)
N(1)-N(2)	1.204(3)
N(2)-N(3)	1.157(3)
N(4)-C(14)	1.335(3)
N(4)-C(11)	1.388(3)
C(1)-C(7)	1.400(3)
C(1)-C(2)	1.423(3)
C(1)-H(1)	0.9300
C(2)-C(3)	1.420(4)
C(2)-C(8)	1.517(4)
C(3)-C(4)	1.414(4)
C(3)-H(3)	0.9300
C(4)-C(5)	1.401(4)
C(4)-H(4)	0.9300
C(5)-C(7)	1.424(4)
C(5)-C(6)	1.500(4)
C(6)-H(6A)	0.9600
C(6)-H(6B)	0.9600
C(6)-H(6C)	0.9600
C(7)-H(7)	0.9300
C(8)-C(10)	1.512(5)
C(8)-C(9)	1.538(4)
C(8)-H(8)	0.9800

C(9)-H(9A)	0.9600
C(9)-H(9B)	0.9600
C(9)-H(9C)	0.9600
C(10)-H(10A)	0.9600
C(10)-H(10B)	0.9600
C(10)-H(10C)	0.9600
C(11)-C(15)	1.398(3)
C(11)-C(12)	1.411(3)
C(12)-C(13)	1.384(3)
C(12)-H(12)	0.9300
C(13)-C(14)	1.410(3)
C(13)-H(13)	0.9300
C(14)-H(14)	0.9300
C(15)-C(16)	1.493(3)
C(16)-C(17)	1.385(3)
C(16)-C(21)	1.407(3)
C(17)-C(18)	1.393(3)
C(17)-H(17)	0.9300
C(18)-C(19)	1.383(4)
C(18)-H(18)	0.9300
C(19)-C(20)	1.379(4)
C(19)-H(19)	0.9300
C(20)-C(21)	1.397(3)
C(20)-H(20)	0.9300
C(22)-H(22A)	0.9600
C(22)-H(22B)	0.9600
C(22)-H(22C)	0.9600
N(4)-Ru(1)-N(1)	86.02(8)
N(4)-Ru(1)-O(1)	77.08(7)
N(1)-Ru(1)-O(1)	86.62(7)
N(4)-Ru(1)-C(4)	93.37(9)
N(1)-Ru(1)-C(4)	127.16(10)
O(1)-Ru(1)-C(4)	144.61(10)
N(4)-Ru(1)-C(3)	98.18(9)
N(1)-Ru(1)-C(3)	164.70(10)

O(1)-Ru(1)-C(3)	108.65(9)
C(4)-Ru(1)-C(3)	38.24(11)
N(4)-Ru(1)-C(2)	126.15(9)
N(1)-Ru(1)-C(2)	145.50(9)
O(1)-Ru(1)-C(2)	89.00(7)
C(4)-Ru(1)-C(2)	69.10(10)
C(3)-Ru(1)-C(2)	38.03(10)
N(4)-Ru(1)-C(5)	115.15(9)
N(1)-Ru(1)-C(5)	96.20(10)
O(1)-Ru(1)-C(5)	167.56(8)
C(4)-Ru(1)-C(5)	37.74(12)
C(3)-Ru(1)-C(5)	68.67(11)
C(2)-Ru(1)-C(5)	81.81(9)
N(4)-Ru(1)-C(7)	151.88(8)
N(1)-Ru(1)-C(7)	89.31(9)
O(1)-Ru(1)-C(7)	130.34(8)
C(4)-Ru(1)-C(7)	67.75(10)
C(3)-Ru(1)-C(7)	80.04(10)
C(2)-Ru(1)-C(7)	67.99(9)
C(5)-Ru(1)-C(7)	37.92(10)
N(4)-Ru(1)-C(1)	164.00(8)
N(1)-Ru(1)-C(1)	109.49(9)
O(1)-Ru(1)-C(1)	99.34(7)
C(4)-Ru(1)-C(1)	80.51(9)
C(3)-Ru(1)-C(1)	67.90(9)
C(2)-Ru(1)-C(1)	37.84(9)
C(5)-Ru(1)-C(1)	68.28(9)
C(7)-Ru(1)-C(1)	37.12(8)
C(15)-O(1)-Ru(1)	114.97(14)
C(21)-O(2)-C(22)	117.2(2)
N(2)-N(1)-Ru(1)	120.43(17)
N(3)-N(2)-N(1)	176.7(3)
C(14)-N(4)-C(11)	107.37(19)
C(14)-N(4)-Ru(1)	138.41(16)
C(11)-N(4)-Ru(1)	114.21(14)
C(7)-C(1)-C(2)	120.1(2)

C(7)-C(1)-Ru(1)	71.00(13)
C(2)-C(1)-Ru(1)	70.21(13)
C(7)-C(1)-H(1)	119.9
C(2)-C(1)-H(1)	119.9
Ru(1)-C(1)-H(1)	131.7
C(3)-C(2)-C(1)	118.7(2)
C(3)-C(2)-C(8)	119.3(2)
C(1)-C(2)-C(8)	121.9(2)
C(3)-C(2)-Ru(1)	70.68(14)
C(1)-C(2)-Ru(1)	71.94(13)
C(8)-C(2)-Ru(1)	127.83(16)
C(4)-C(3)-C(2)	119.9(2)
C(4)-C(3)-Ru(1)	69.63(15)
C(2)-C(3)-Ru(1)	71.29(13)
C(4)-C(3)-H(3)	120.1
C(2)-C(3)-H(3)	120.1
Ru(1)-C(3)-H(3)	131.9
C(5)-C(4)-C(3)	121.9(2)
C(5)-C(4)-Ru(1)	72.96(15)
C(3)-C(4)-Ru(1)	72.12(14)
C(5)-C(4)-H(4)	119.1
C(3)-C(4)-H(4)	119.1
Ru(1)-C(4)-H(4)	128.1
C(4)-C(5)-C(7)	117.6(2)
C(4)-C(5)-C(6)	122.1(3)
C(7)-C(5)-C(6)	120.2(3)
C(4)-C(5)-Ru(1)	69.30(14)
C(7)-C(5)-Ru(1)	71.21(13)
C(6)-C(5)-Ru(1)	129.24(19)
C(5)-C(6)-H(6A)	109.5
C(5)-C(6)-H(6B)	109.5
H(6A)-C(6)-H(6B)	109.5
C(5)-C(6)-H(6C)	109.5
H(6A)-C(6)-H(6C)	109.5
H(6B)-C(6)-H(6C)	109.5
C(1)-C(7)-C(5)	121.7(2)

C(1)-C(7)-Ru(1)	71.88(13)
C(5)-C(7)-Ru(1)	70.88(13)
C(1)-C(7)-H(7)	119.2
C(5)-C(7)-H(7)	119.2
Ru(1)-C(7)-H(7)	130.9
C(10)-C(8)-C(2)	114.4(3)
C(10)-C(8)-C(9)	110.8(3)
C(2)-C(8)-C(9)	108.2(2)
C(10)-C(8)-H(8)	107.8
C(2)-C(8)-H(8)	107.8
C(9)-C(8)-H(8)	107.8
C(8)-C(9)-H(9A)	109.5
C(8)-C(9)-H(9B)	109.5
H(9A)-C(9)-H(9B)	109.5
C(8)-C(9)-H(9C)	109.5
H(9A)-C(9)-H(9C)	109.5
H(9B)-C(9)-H(9C)	109.5
C(8)-C(10)-H(10A)	109.5
C(8)-C(10)-H(10B)	109.5
H(10A)-C(10)-H(10B)	109.5
C(8)-C(10)-H(10C)	109.5
H(10A)-C(10)-H(10C)	109.5
H(10B)-C(10)-H(10C)	109.5
N(4)-C(11)-C(15)	114.98(19)
N(4)-C(11)-C(12)	109.09(19)
C(15)-C(11)-C(12)	135.9(2)
C(13)-C(12)-C(11)	106.2(2)
C(13)-C(12)-H(12)	126.9
C(11)-C(12)-H(12)	126.9
C(12)-C(13)-C(14)	107.2(2)
C(12)-C(13)-H(13)	126.4
C(14)-C(13)-H(13)	126.4
N(4)-C(14)-C(13)	110.1(2)
N(4)-C(14)-H(14)	124.9
C(13)-C(14)-H(14)	124.9
O(1)-C(15)-C(11)	118.72(19)

O(1)-C(15)-C(16)	117.3(2)
C(11)-C(15)-C(16)	123.9(2)
C(17)-C(16)-C(21)	119.6(2)
C(17)-C(16)-C(15)	119.5(2)
C(21)-C(16)-C(15)	120.7(2)
C(16)-C(17)-C(18)	120.7(2)
C(16)-C(17)-H(17)	119.6
C(18)-C(17)-H(17)	119.6
C(19)-C(18)-C(17)	119.1(2)
C(19)-C(18)-H(18)	120.5
C(17)-C(18)-H(18)	120.5
C(20)-C(19)-C(18)	121.4(2)
C(20)-C(19)-H(19)	119.3
C(18)-C(19)-H(19)	119.3
C(19)-C(20)-C(21)	119.7(2)
C(19)-C(20)-H(20)	120.1
C(21)-C(20)-H(20)	120.1
O(2)-C(21)-C(20)	125.1(2)
O(2)-C(21)-C(16)	115.4(2)
C(20)-C(21)-C(16)	119.5(2)
O(2)-C(22)-H(22A)	109.5
O(2)-C(22)-H(22B)	109.5
H(22A)-C(22)-H(22B)	109.5
O(2)-C(22)-H(22C)	109.5
H(22A)-C(22)-H(22C)	109.5
H(22B)-C(22)-H(22C)	109.5

Symmetry transformations used to generate equivalent atoms:

Table 4. Anisotropic displacement parameters ($Å^2$ x 10^3) for 5. The anisotropic displacement factor exponent takes the form: $-2\pi^2[h^2 a^{*2}U^{11} + ... + 2 h k a^* b^* U^{12}]$

	U^{11}	U^{22}	U^{33}	U^{23}	U^{13}	U^{12}
Ru(1)	18(1)	20(1)	20(1)	1(1)	9(1)	3(1)
O(1)	25(1)	24(1)	28(1)	4(1)	15(1)	4(1)
O(2)	48(1)	23(1)	44(1)	-5(1)	27(1)	-6(1)
N(1)	23(1)	31(1)	33(1)	-8(1)	10(1)	-1(1)
N(2)	27(1)	22(1)	27(1)	-2(1)	9(1)	-1(1)
N(3)	44(1)	34(1)	44(1)	-10(1)	26(1)	-1(1)
N(4)	19(1)	20(1)	23(1)	1(1)	9(1)	2(1)
C(1)	23(1)	23(1)	27(1)	1(1)	12(1)	8(1)
C(2)	30(1)	23(1)	32(1)	-5(1)	18(1)	2(1)
C(3)	28(1)	45(1)	24(1)	-10(1)	10(1)	6(1)
C(4)	36(1)	50(2)	25(1)	9(1)	18(1)	19(1)
C(5)	34(1)	29(1)	46(2)	11(1)	30(1)	11(1)
C(6)	61(2)	36(2)	100(3)	26(2)	60(2)	12(1)
C(7)	19(1)	26(1)	38(1)	-3(1)	15(1)	3(1)
C(8)	40(2)	24(1)	65(2)	-10(1)	31(2)	-5(1)
C(9)	75(3)	31(2)	97(3)	-25(2)	49(2)	-10(2)
C(10)	82(3)	28(1)	75(2)	14(2)	51(2)	5(2)
C(11)	20(1)	20(1)	25(1)	-2(1)	11(1)	-1(1)
C(12)	19(1)	27(1)	35(1)	-4(1)	12(1)	1(1)
C(13)	23(1)	26(1)	30(1)	0(1)	5(1)	5(1)
C(14)	28(1)	22(1)	23(1)	2(1)	9(1)	1(1)
C(15)	22(1)	19(1)	26(1)	-2(1)	12(1)	-1(1)
C(16)	23(1)	25(1)	28(1)	2(1)	13(1)	-1(1)
C(17)	38(2)	28(1)	41(1)	-1(1)	26(1)	-1(1)
C(18)	49(2)	39(1)	44(2)	-4(1)	33(1)	-1(1)
C(19)	37(2)	43(2)	40(1)	11(1)	24(1)	0(1)
C(20)	26(1)	28(1)	37(1)	7(1)	13(1)	-2(1)
C(21)	22(1)	25(1)	27(1)	3(1)	9(1)	0(1)
C(22)	44(2)	23(1)	48(2)	-1(1)	14(1)	-2(1)

Table 5. Hydrogen coordinates (x 10^4) and isotropic displacement parameters ($Å^2$x 10^3) for 5.

	x	y	z	U(eq)
H(1)	5344	7418	5537	28
H(3)	7299	7324	8340	39
H(4)	6868	5051	8669	42
H(6A)	6015	2866	8001	86
H(6B)	5279	2912	6905	86
H(6C)	5159	3543	7892	86
H(7)	4903	5200	5916	32
H(8)	7335	8909	6872	48
H(9A)	6808	11098	7187	94
H(9B)	6750	9955	7995	94
H(9C)	5952	10372	7022	94
H(10A)	6487	8583	5091	83
H(10B)	6690	10218	5377	83
H(10C)	5817	9593	5243	83
H(12)	9625	4183	7081	32
H(13)	9443	2604	8442	33
H(14)	8003	2994	8371	29
H(17)	8604	4386	4643	39
H(18)	9166	5208	3463	47
H(19)	9447	7643	3408	44
H(20)	9186	9263	4511	36
H(22A)	9295	10329	6134	59
H(22B)	8509	10669	6391	59
H(22C)	8425	10539	5223	59

Table 3. Hydrogen bonding distances and angles for **2-5** (Å and °).

D-H...A	d(D-H)	d(H...A)	d(D...A)	<(DHA)
		**2**		
C1-H1B...Cl1	0.9800	2.8100	3.4603(19)	125.00
C3-H3...O1#1	0.9500	2.4900	3.1721(19)	128.00
C4-H4...Cl1#1	0.9500	2.8100	3.7475(16)	170.00
C8-H...O2	1.0000	2.5100	3.366(2)	143.00
		**3**		
C13-H13...Cl1	0.9300	2.6900	3.545(8)	153.00
C33-H33...Cl1	0.9300	2.6900	3.415(8)	135.00
C39-H39...O2	0.9300	2.5300	2.988(9)	111.00
		**4**		
C20-H20...O1	0.9800	2.5400	3.425(7)	150.00
		**5**		
C6-H6A...N3#2	0.9600	2.5900	3.488(5)	155.00
C7-H7...N1#3	0.9300	2.5400	3.226(3)	131.00
C10-H10A...O1	0.9600	2.5500	3.270(4)	132.00

Symmetry transformations used to generate equivalent atoms:

#1 1-x,1-y,-z; #2 x,1/2-y,1/2+z; #3 1-x,1-y,1-z
